# Crystal structures of three bicyclic carbohydrate derivatives

**DOI:** 10.1107/S2056989016018727

**Published:** 2016-11-29

**Authors:** Uwe Schilde, Alexandra Kelling, Sumaira Umbreen, Torsten Linker

**Affiliations:** a Universität Potsdam, Institut für Chemie, Anorganische Chemie, Karl-Liebknecht-Strasse 24-25, D-14476 Potsdam, Germany

**Keywords:** crystal structure, carbohydrate deriv­atives, conformation, configuration

## Abstract

The title compounds, [(1*R*,3*R*,4*R*,5*R*,6*S*)-4,5-bis­(acet­yloxy)-7-oxo-2-oxabi­cyclo[4.2.0]octan-3-yl]methyl acetate, C_14_H_18_O_8_, (I), [(1*S*,4*R*,5*S*,6*R*)-5-acet­yloxy-7-hy­droxy­imino-2-oxobi­cyclo­[4.2.0]octan-4-yl acetate, C_11_H_15_NO_6_, (II), and [(3a*R*,5*R*,6*R*,7*R*,7a*S*)-6,7-bis­(acet­yloxy)-2-oxo­octa­hydro­pyrano[3,2-*b*]pyrrol-5-yl]methyl acetate, C_14_H_19_NO_8_, (III), are stable bicyclic carbohydrate derivatives. They can easily be synthesized in a few steps from commercially available glycals. As a result of the ring strain from the four-membered rings in (I) and (II), the conformations of the carbohydrates deviate strongly from the ideal chair form. Compound (II) occurs in the boat form. In the five-membered lactam (III), on the other hand, the carbohydrate adopts an almost ideal chair conformation. As a result of the distortion of the sugar rings, the configurations of the three bicyclic carbohydrate derivatives could not be determined from their NMR coupling constants. From our three crystal structure determinations, we were able to establish for the first time the absolute configurations of all new stereocenters of the carbohydrate rings.

## Abstract 2

The structures of three bicyclic carbohydrates derivatives containing cyclo­butanone or cyclo­lactame beside the pyran­ose ring are reported and the conformation and configuration established.

## Chemical context   

Bicyclic carbohydrate derivatives have become attractive as inhibitors of glycoside hydro­lases (Lahiri *et al.*, 2013 ▸). In particular, the enzyme *O*-GlcNAcase (OGA) is a promising target for such small-mol­ecule inhibitors, since the level of *O*-GlcNAc in our body influences diseases such as Alzheim­er’s (Yuzwa *et al.*, 2012 ▸) or cancer (Ma & Vosseller, 2013 ▸). However, the synthesis of bicyclic carbohydrate derivatives is usually a multi-step procedure. During our studies on the syntheses of carbohydrate analogs (Yin & Linker, 2012 ▸), we developed an easy entry to such compounds by radical additions to commercially available glycals (Linker *et al.*, 1997 ▸).
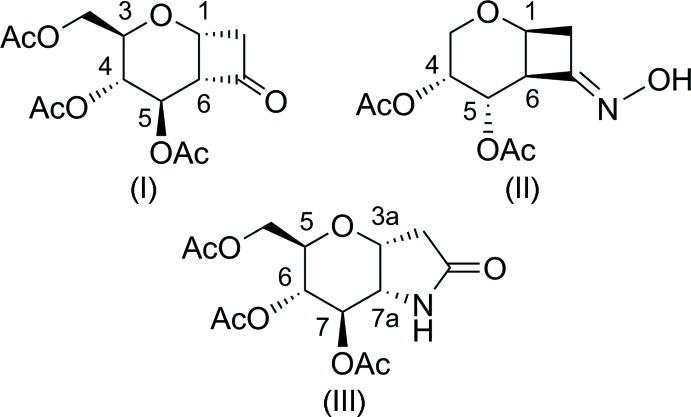


More recently, we became inter­ested in cyclo­additions to glycals, affording bicyclic carbohydrate derivatives in one single step (Linker & Umbreen, 2012 ▸; Umbreen & Linker, 2015 ▸). Using this procedure the products (I), (II), and (III) were isolated in good yields in analytically pure form by column chromatography. However, their conformations and absolute configurations could not be determined by NMR spectroscopy, due to distortion of the sugar rings. Herein we report their crystal structures, which establish their absolute configurations and conformations in the solid state.

## Structural commentary   

**Figure 1:**
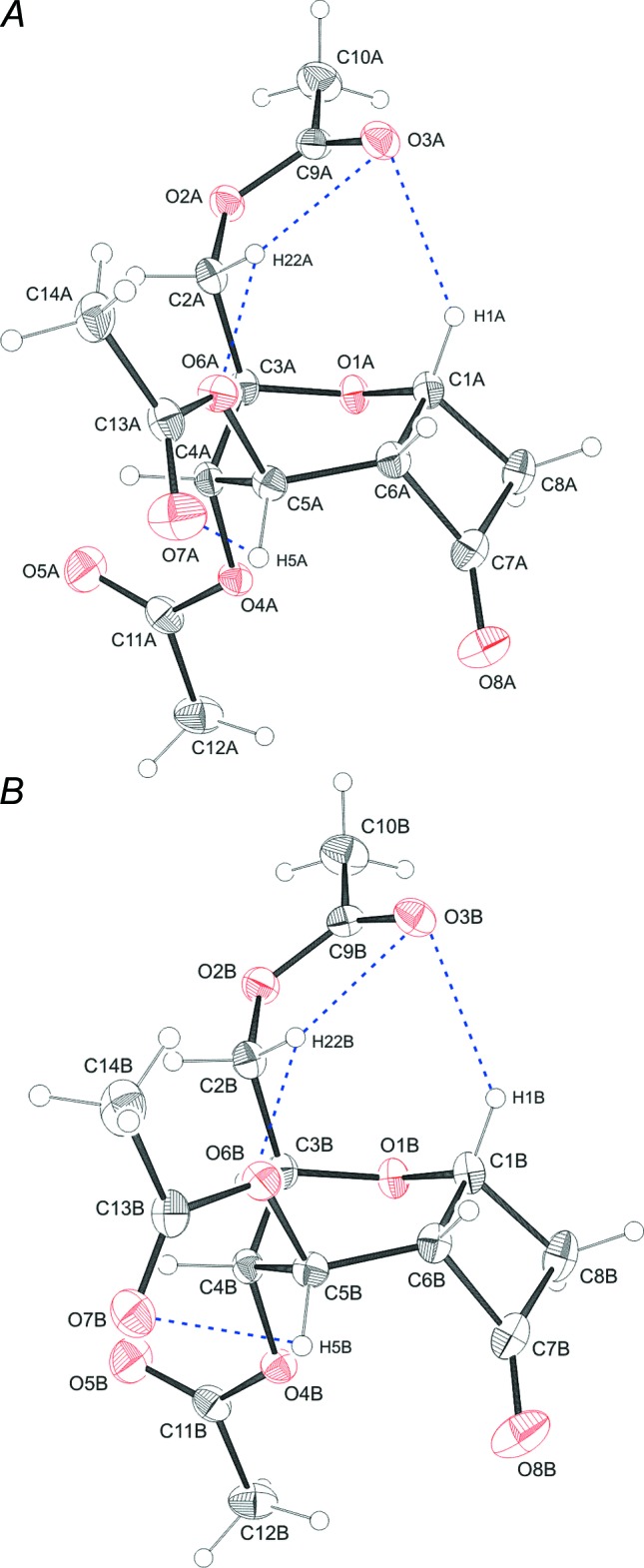
The mol­ecular structure of the two independent mol­ecules (*A* and *B*) of compound (I), showing the atom labelling. Displacement ellipsoids are drawn at the 30% probability level. H atoms are shown as small spheres of arbitrary radius and hydrogen bonds are shown as blue dashed lines.

Crystals of (I) and (II) are monoclinic, space group *P*2_1_. There are two mol­ecules in the asymmetric unit of compound (I), which show small conformational differences, especially the two acet­yloxy substituents in the 4- and 5-positions (Fig. 1 ▸). The largest differences occur for the corresponding torsion angles C6—C5—O6—C13 [mol­ecule *A* −118.6 (2)°, mol­ecule *B* −147.5 (2)°]. The conformation of the pyran­ose rings deviates from the ideal chair. The puckering amplitudes and smallest displacement parameters for mol­ecules *A* and *B* are *q* = 0.467 (2)/0.473 (3) Å, *θ* = 151.9 (4)/150.7 (4)° and *φ* = 114.1 (6)/114.3 (6)°. The main feature is the absolute configuration of the new stereocenters being 1*R* and 6*S*. Surprisingly, the acet­yloxy substituents are positioned axially, in contrast to the usual d-*gluco* arrangement. Obviously the cyclo­butanone ring, with its bis­ectional positioned (C1—C8) and axial bonds (C6—C7) – in relation to the pyran­ose ring – enforces a flipping of the chair from ^4^ *C*_1_ into ^1^ *C*_4_. The cyclo­butane ring is almost planar [maximum deviation from the best plane of C7 = 0.0762 (15) Å in *A* and 0.0815 (15) Å in *B*] and can be described by the dihedral angles, forming by folding along the C6⋯C8 and C1⋯C7 line, between the planes C1–C6–C8/C6–C7–C8 [*A* 15.5 (2)°, *B* 17.0 (2)°] and C1–C6–C7/C1–C7–C8 [*A* 15.5 (2)°, *B* 16.6 (2)°]. The deviation of the carbonyl O atoms (O8*A*/O8*B*) from the mean plane of the pyran ring are 0.253 (5) and 0.303 (6) Å in mol­ecules *A* and *B*, respectively. The dihedral angles between the pyran­ose rings and the cyclo­butane rings are 61.3 (1) and 62.1 (1)° for mol­ecules *A* and *B*, respectively. Four non-classical intra­molecular hydrogen bonds for each of the both mol­ecules can be observed (see Fig. 1 ▸ and Table 1 ▸).

**Figure 2:**
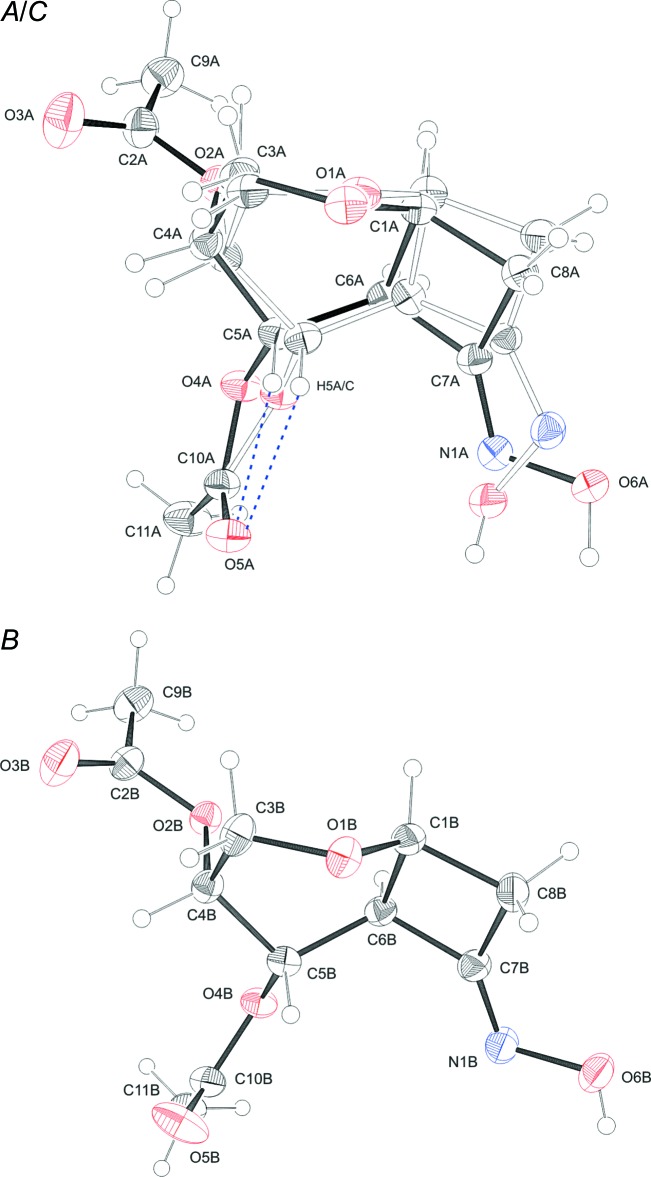
The mol­ecular structure of the two independent mol­ecules (*A* and *B*) of compound (II), showing the atom labelling, rendering the disorder of mol­ecule *A* [occupancy ratio = 0.802 (7):0.198 (7)] with open bonds for the minor component. Displacement ellipsoids are drawn at the 30% probability level. H atoms are shown as small spheres of arbitrary radius and hydrogen bonds are shown as blue dashed lines.

Compound (II) also crystallizes with two mol­ecules in the asymmetric unit. Mol­ecule *A* is disordered (the minor component is labelled with the letter *C*; for details - see *Refinement* section). Mol­ecules *A*, *B* and *C* mainly differ in the torsion angles C10—O4—C5—C6 [*A* 115.6 (4)°, *B*: 149.4 (2)°] and O4—C5—C6—C1 [*A* 165.1 (5)°, *B* 167.6 (2)°, *C* 155 (2)°] of the acet­yloxy substituents in the 5-position (Fig. 2 ▸). The pyran­ose rings adopt a twisted-boat conformation, characterized by the puckering parameters *q* = 0.755 (8)/0.763 (3)/0.75 (3) Å, *θ* = 90.9 (6)/91.0 (2)/91 (2)° and *φ* = 12.6 (6)/12.7 (2)/28 (3)° for mol­ecules *A*, *B* and *C*, and not the usual chair conformation. This arrangement is caused by the cyclo­butane ring with the C1—C8 and C6—C7 bonds, which are bis­ectional related to the arabinose ring. The absolute configuration on the stereocenters of the shared ring atoms is C1*S* and C6*R*. The cyclo­butane rings are almost planar with maximum deviations from the best plane of 0.045 (3) Å (C7*A*), 0.039 (1) Å (C7*B*) and 0.072 (12) Å (C7*C*). The nitro­gen atoms deviate marginally from these planes [N1*A* −0.224 (9) Å, N1*B* 0.199 (4) Å, N1*C* 0.30 (4) Å. The dihedral angles within the four-membered rings between C1/C6/C8 and C6/C7/C8 are 9.4 (5)° (*A*), 8.2 (2)°, (*B*) and 15 (2)° (*C*), and between C1/C6/C7 and C1/C7/C8 they are 9.0 (5)° (*A*), 7.9 (3)° (*B*) and 14 (2)° (*C*). The hydroxyl group of the oxime substit­uent can adopt two different configurations. Mol­ecule *B* exhibits an *E* configuration. For disordered mol­ecules *A* and *C*, the *E*/*Z* ratio of the isomers is 0.802 (7):0.198 (7). Thus, the major component (*A*) is *E* configured, with the hydroxyl group pointing away from the six-membered ring. In the minor *Z* isomer (*C*), the hydroxyl group exhibits a sterically unfavourable inter­action with the carbohydrate ring. An intra­molecular hydrogen bond between C5*A*/C5*C* and O5*A* is observed (Fig. 2 ▸, Table 2 ▸).

**Figure 3:**
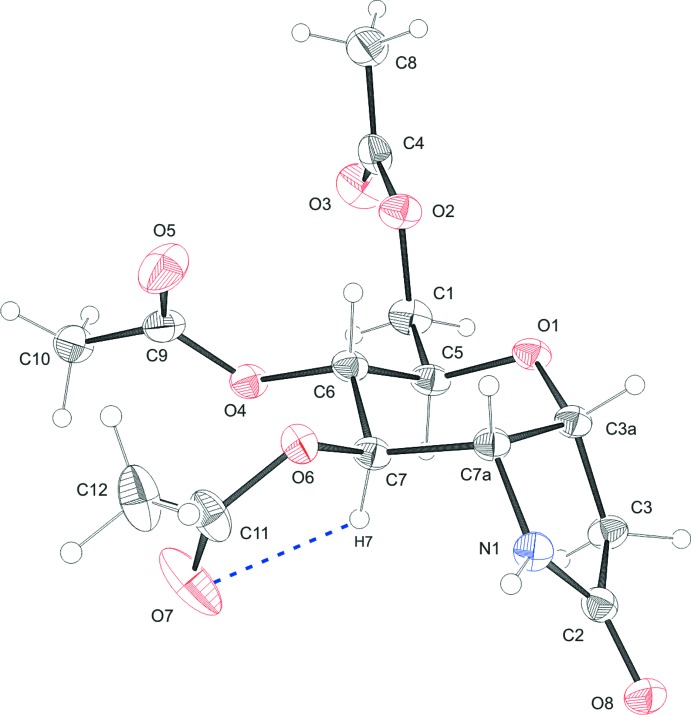
The mol­ecular structure of compound (III), showing the atom labelling. Displacement ellipsoids are drawn at the 30% probability level. H atoms are shown as small spheres of arbitrary radius and hydrogen bonds are shown as blue dashed lines.

Compound (III) contains one mol­ecule in the asymmetric unit (Fig. 3 ▸). The new stereocenter at C7a obtained during synthesis is *S* configured. The six-membered and the five-membered rings are fused in the *cis* configuration. The C3a—C3 bond is axial and the C7a—N1 bond is bis­ectionally positioned with respect to the pyran­ose ring. The pyran­ose ring exhibits a strongly distorted chair conformation, with puckering parameters *q* = 0.555 (3) Å, *θ* = 20.4 (3)° and *φ* = 267.9 (9)°. The usual d-*gluco* configuration in the chair form ^4^ *C*_1_ is found, in contrast to (I). The pyrrolidonyl ring is in an envelope conformation, closed puckering on C3a with a maximum deviation for that atom of 0.466 (5) Å from the plane formed by N1, C2, C3 and C7a. An intra­molecular hydrogen bond is observed between C7 and O7 (Fig. 3 ▸, Table 3 ▸). In (I) and (II), the correct absolute configuration was assigned in agreement with the known chirality of the glycal precursors. Compound (III) was synthesized from (I) and thus its absolute configuration is known as well.

## Supra­molecular features   

**Figure 4:**
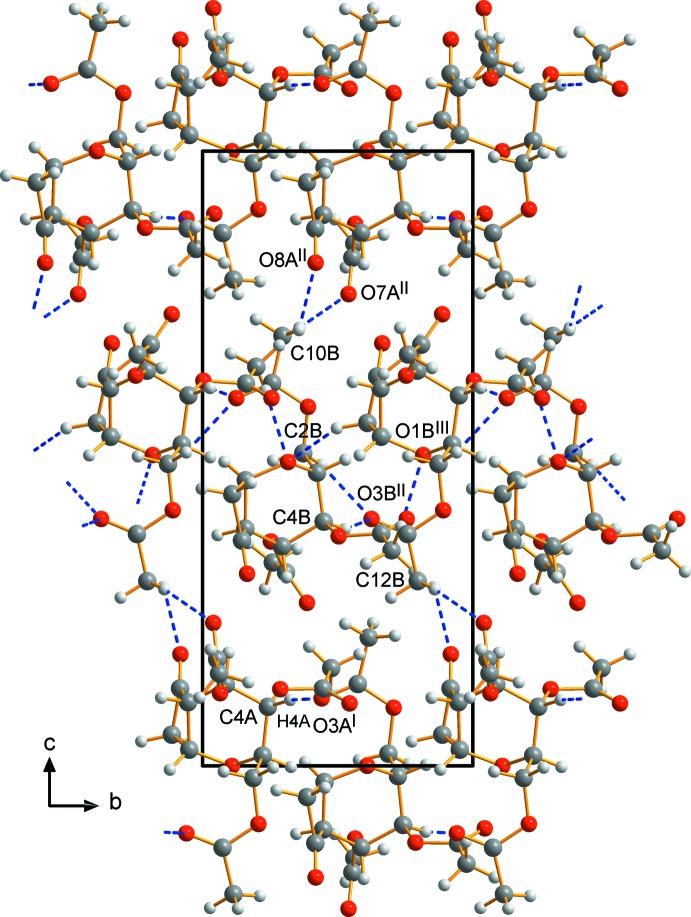
Part of the crystal of (I), with inter­molecular hydrogen bonds shown as blue dashed lines. The view is along the *a* axis.

The crystal packing of (I) features weak non-classical C—H⋯O hydrogen bonds, which are illustrated in Fig. 4 ▸ and listed in Table 1 ▸. The *A* mol­ecules are hydrogen-bonded *via* C4*A*—H4*A*⋯O3*A* ^i^ inter­actions screwing around the *b*-axis direction (Fig. 4 ▸). Between two infinite chains of *A* mol­ecules (above and below in in Fig. 4 ▸) , the *B* mol­ecules are located, again forming a screw *via* three hydrogen bonds (C2*B*—H21*B*⋯O3*B* ^ii^, C4*B*—H4*B*⋯O3*B* ^ii^ and C12*B*—H125⋯O1*B* ^iii^). The *A* and *B* mol­ecules are linked by two further hydrogen bonds (C10*B*—H104⋯O8*A* ^ii^ and C10*B*—H106⋯O7*A* ^ii^).

**Figure 5:**
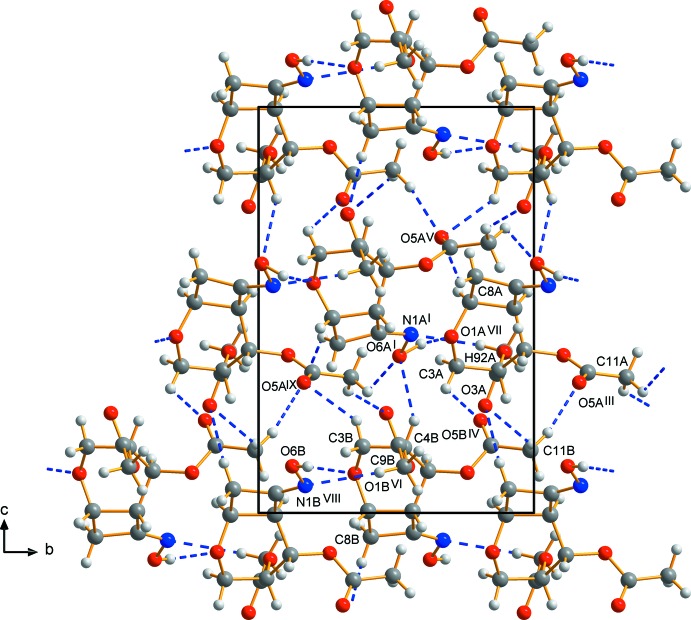
Part of the crystal of (II), with inter­molecular hydrogen bonds shown as blue dashed lines. The minor disorder component has been omitted for clarity. The view is along the *a* axis.

The crystal packing of (II) is similar to that of (I). Chains consisting only of *A* mol­ecules are in an alternating arrangement with those consisting only of *B* mol­ecules, both screwing along the *b*-axis direction (Fig. 5 ▸). In contrast to (I), more inter­molecular hydrogen bonds can be observed. Strong hydrogen bonds occur between the OH groups and the oxygen atoms of the pyran­ose rings within each chain. Weak C—H⋯O and C—H⋯N hydrogen bonds act as linkers between the chains of mol­ecules. The chains are further connected *via* a large number of hydrogen bonds. Hydrogen bond geometries are summarized in Table 2 ▸.

**Figure 6:**
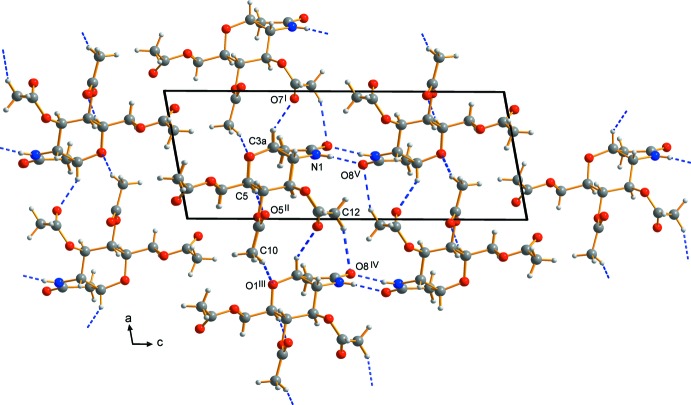
Part of the crystal of (III), with inter­molecular hydrogen bonds shown as blue dashed lines. The view is along the *b* axis.

In the crystal packing of (III), mol­ecules are linked *via* weak C—H⋯O hydrogen bonds running along the *a*-axis direction. The chains formed this way are connected in a pairwise fashion by strong N1—H1*A*⋯O8 bonds along *c* (see Fig. 6 ▸ and Table 3 ▸).

## Database survey   

For structures containing the 2-oxabi­cyclo­[4.2.0]octane unit, see Tsao & Isobe (2010 ▸) and Li *et al.* (2012 ▸). For a structure with the octa­hydro­pyrano[3,2*b*]pyrrol-2-one moiety, see Nastopoulos *et al.* (1997 ▸).

## Synthesis and crystallization   

**Cyclo­butanone (I)** was synthesized from tri-*O*-acetyl-d-glucal, commercially available or obtained by the procedure of Ferrier (1965 ▸). Tri­chloro­acetyl chloride (2.18 g, 10 mmol) in diethyl ether (12 mL) was added to a mixture of zinc–copper couple (3.87 g, 30 mmol) and tri-*O*-acetyl-d-glucal (1.36 g, 5 mmol) in dry diethyl ether (30 mL) at room temperature over 30 min under an N_2_ atmosphere. After completion of the reaction (TLC control), zinc dust (3.27 g, 50 mmol) was added at 273 K. Acetic acid (13 mL) was added within 10 min and the reaction mixture was stirred for 60 min. The reaction mixture was diluted with diethyl ether (60 mL) and the insoluble materials were filtered off through Celite, which was washed with diethyl ether (5 × 50 mL) and methanol (50 mL). The filtrate was extracted with (3 × 100 mL) water. The organic layer was dried over MgSO_4_ and concentrated *in vacuo*. The resulting residue was purified by column chromatography (hexa­ne/ethyl acetate 5:1) to afford pure cyclo­butanone (I) (1.41 g, 90%). Single crystals suitable for X-ray diffraction were prepared by slow evaporation of a solution of (I) in ethanol at room temperature.

**Oxime (II)** was synthesized from di-*O*-acetyl-d-arabinal, obtained by the procedure of Ferrier (1965 ▸). Starting from di-*O*-acetyl-d-arabinal (1.0 g, 5.0 mmol) the corresponding cyclo­butanone was synthesized as described above and isolated by column chromatography (hexa­ne/ethyl acetate 5:1) in 83% yield. 242 mg (1.0 mmol) of this cyclo­butanone was dissolved in ethanol (2 mL) and then added to a solution of sodium acetate (246 mg, 3.0 mmol) and hydroxyl­amine hydro­chloride (208 mg, 3.0 mmol) in water (2 mL). The reaction mixture was stirred at 327 K for 2 h and then for 1 h at room temperature. The reaction mixture was washed with water (30 mL) and extracted with CH_2_Cl_2_ (3 × 50 mL). The organic layers were combined, dried over MgSO_4_, filtered and concentrated *in vacuo*. The oxime (II) was directly recrystallized from ethanol solution, whereupon single crystals suitable for X-ray diffraction were obtained.

**Lactam (III)** was synthesized from cyclo­butanone (I) (314 mg, 1 mmol). This cyclo­butanone was dissolved in ethanol (2 mL) and then added to a solution of sodium acetate (246 mg, 3.0 mmol) and hydroxyl­amine hydro­chloride (208 mg, 3.0 mmol) in water (2 mL). The reaction mixture was stirred at 327 K for 2 h and then for 1 h at room temperature. The reaction mixture was washed with water (30 mL) and extracted with CH_2_Cl_2_ (3 × 50 mL). The organic layers were combined, dried over MgSO_4_, filtered and concentrated *in vacuo*. Thionyl chloride (217.5 µL, 3.0 mmol) was added to a solution of the crude oxime in 1,4-dioxane (4 mL), and stirred for 10 min at room temperature. The reaction was quenched with saturated aqueous NaHCO_3_ (50 mL), and extracted with EtOAc (3 × 100 mL). The organic extracts were washed with brine, dried over MgSO_4_, and concentrated *in vacuo*. The residue was purified by column chromatography (hexa­ne/ethyl acetate 1:4) to afford the lactam in analytically pure form (244 mg, 74%). Single crystals suitable for X-ray diffraction were prepared by slow evaporation of a solution of (III) in ethanol at room temperature.

## Refinement   

In compound (II), disorder was observed for mol­ecule *A*, caused by flipping of the N—OH group. That disorder also causes disorder of the nearby ring atoms. Therefore the ring atoms of both the five- and six-membered rings were included in the disorder (but the OAc groups were left out). The geometry of the minor component was restrained to be similar to that of the major one with SAME, SADI and SIMU 0.01 restraints. The refinement of the occupation factors revealed an occupation ratio of 0.802 (7)/0.198 (7) for the two disordered components (see Fig. 2 ▸). H atoms in the structures of (I), (III) and the ordered and major components of (II) were located from difference Fourier maps and refined as riding with *U*_iso_(H) = 1.2*U*_eq_(C) with the exception of methyl hydrogen atoms, which were placed in their expected positions with HFIX 137 and refined with *U*_iso_(H) = 1.5*U*_eq_(C). For the minor disordered component in compound (II), all H atoms were placed in their expected positions with C—H distances of 0.99 and 0.98 for CH and CH_2_ groups (HFIX 13 and 23) and 0.83 Å for OH groups (HFIX 147), and with *U*_iso_(H) = 1.2*U*_eq_(C) and 1.5*U*_eq_(O). Crystal data, data collection and structure refinement details are summarized in Table 4 ▸.

## Supplementary Material

Crystal structure: contains datablock(s) I, II, III, global. DOI: 10.1107/S2056989016018727/zl2684sup1.cif

Structure factors: contains datablock(s) I. DOI: 10.1107/S2056989016018727/zl2684Isup2.hkl

Structure factors: contains datablock(s) II. DOI: 10.1107/S2056989016018727/zl2684IIsup3.hkl

Structure factors: contains datablock(s) III. DOI: 10.1107/S2056989016018727/zl2684IIIsup4.hkl

Supporting information file. DOI: 10.1107/S2056989016018727/zl2684Isup5.cml

Supporting information file. DOI: 10.1107/S2056989016018727/zl2684IIsup6.cml

Supporting information file. DOI: 10.1107/S2056989016018727/zl2684IIIsup7.cml

CCDC references: 1518715, 1518714, 1518713

Additional supporting information:  crystallographic information; 3D view; checkCIF report

## Figures and Tables

**Table 1:** Hydrogen-bond geometry (Å, °) for (I)

*D*—H⋯*A*	*D*—H	H⋯*A*	*D*⋯*A*	*D*—H⋯*A*
C1*A*—H1*A*⋯O3*A*	0.98 (3)	2.41 (3)	3.184 (3)	135 (2)
C2*A*—H22*A*⋯O3*A*	0.93 (3)	2.31 (3)	2.697 (3)	104 (2)
C2*A*—H22*A*⋯O6*A*	0.93 (3)	2.46 (3)	2.876 (3)	107 (2)
C5*A*—H5*A*⋯O7*A*	0.95 (3)	2.27 (3)	2.701 (3)	106.8 (19)
C1*B*—H1*B*⋯O3*B*	1.02 (3)	2.44 (3)	3.237 (3)	134 (2)
C2*B*—H22*B*⋯O3*B*	0.92 (4)	2.34 (3)	2.699 (3)	103 (2)
C2*B*—H22*B*⋯O6*B*	0.92 (4)	2.52 (3)	2.929 (3)	108 (2)
C5*B*—H5*B*⋯O7*B*	0.95 (3)	2.34 (3)	2.688 (3)	101 (2)
C4*A*—H4*A*⋯O3*A* ^i^	0.95 (3)	2.44 (3)	3.333 (3)	157 (2)
C2*B*—H21*B*⋯O3*B* ^ii^	1.00 (4)	2.49 (4)	3.400 (3)	150 (2)
C4*B*—H4*B*⋯O3*B* ^ii^	0.98 (3)	2.46 (3)	3.338 (3)	148 (2)
C10*B*—H104⋯O8*A* ^ii^	0.97	2.55	3.306 (4)	135
C10*B*—H106⋯O7*A* ^ii^	0.97	2.52	3.472 (4)	169
C12*B*—H125⋯O1*B* ^iii^	0.97	2.52	3.476 (3)	167

Symmetry codes: (i) 
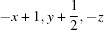
; (ii) 
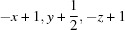
; (iii) 
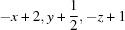
.

**Table 2:** Hydrogen-bond geometry (Å, °) for (II)

*D*—H⋯*A*	*D*—H	H⋯*A*	*D*⋯*A*	*D*—H⋯*A*
C5*A*—H5*A*⋯O5*A*	0.98 (4)	2.21 (4)	2.691 (6)	109 (3)
C11*B*—H115⋯O3*A*	0.97	2.65	3.417 (4)	136
C9*A*—H92*A*⋯N1*A* ^i^	0.97	2.54	3.482 (6)	163
C4*B*—H4*B*⋯O6*A* ^i^	0.99	2.64	3.407 (3)	135
C9*A*—H92*A*⋯O6*C* ^i^	0.97	2.15	3.115 (15)	175
C1*C*—H1*C*⋯O4*C* ^i^	0.99	2.56	3.52 (4)	165
C11*A*—H112⋯O3*B* ^ii^	0.97	2.59	3.413 (4)	142
C11*B*—H116⋯O5*A* ^iii^	0.97	2.36	3.312 (3)	169
C3*A*—H32*A*⋯O5*B* ^iv^	1.01 (4)	2.50 (4)	3.176 (12)	124 (3)
C3*C*—H32*C*⋯O5*B* ^iv^	0.98	2.25	3.05 (5)	138
C8*A*—H82*A*⋯O5*A* ^v^	0.92 (5)	2.73 (5)	3.329 (5)	124 (3)
C8*C*—H82*C*⋯O5*A* ^v^	0.98	2.62	3.27 (2)	123
C8*C*—H82*C*⋯O6*C* ^v^	0.98	2.50	3.32 (3)	141
O6*B*—H62⋯O1*B* ^vi^	0.89 (5)	1.96 (5)	2.852 (3)	175 (4)
O6*A*—H61*A*⋯O1*A* ^vii^	0.90 (5)	1.86 (5)	2.757 (9)	175 (5)
C11*A*—H113⋯O6*A* ^vii^	0.97	2.61	3.199 (4)	120
O6*C*—H61*C*⋯O1*C* ^vii^	0.83	2.35	2.96 (5)	131
C8*B*—H81*B*⋯O3*A* ^viii^	0.98	2.62	3.498 (4)	150
C9*B*—H92*B*⋯N1*B* ^viii^	0.97	2.69	3.635 (4)	164
C3*B*—H32*B*⋯O5*A* ^ix^	0.98	2.61	3.430 (3)	142
C8*B*—H82*B*⋯N1*B* ^x^	0.98	2.67	3.569 (4)	152

Symmetry codes: (i) 
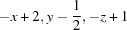
; (ii) 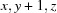; (iii) 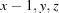; (iv) 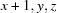; (v) 
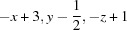
; (vi) 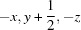; (vii) 
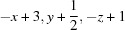
; (viii) 
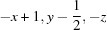
; (ix) 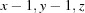; (x) 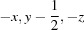.

**Table 3:** Hydrogen-bond geometry (Å, °) for (III)

*D*—H⋯*A*	*D*—H	H⋯*A*	*D*⋯*A*	*D*—H⋯*A*
C7—H7⋯O7	0.94 (4)	2.32 (3)	2.695 (4)	103 (3)
C3*A*—H3*A*⋯O7^i^	0.97 (4)	2.42 (4)	3.243 (4)	143 (3)
C5—H5⋯O5^ii^	0.98 (4)	2.27 (4)	3.230 (4)	167 (3)
C10—H10*A*⋯O1^iii^	0.97	2.47	3.386 (4)	158
C12—H12*C*⋯O8^iv^	0.97	2.58	3.468 (4)	152
N1—H1⋯O8^v^	0.87 (4)	1.96 (4)	2.826 (3)	174 (4)

Symmetry codes: (i) 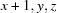; (ii) 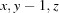; (iii) 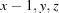; (iv) 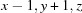; (v) 
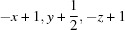
.

**Table 4:** Experimental details

	(I)	(II)	(III)
Crystal data			
Chemical formula	C_14_H_18_O_8_	C_11_H_15_NO_6_	C_14_H_19_NO_8_
*M*_r_	314.28	257.24	329.30
Crystal system, space group	Monoclinic, *P*2_1_	Monoclinic, *P*2_1_	Monoclinic, *P*2_1_
Temperature (K)	210	210	210
*a*, *b*, *c* (Å)	9.5538 (6), 8.3655 (3), 19.1395 (10)	8.9910 (3), 9.6231 (5), 14.4915 (6)	7.0784 (5), 6.1454 (3), 18.5176 (12)
β (°)	96.837 (4)	101.742 (3)	100.476 (5)
*V* (Å^3^)	1518.80 (14)	1227.59 (9)	792.08 (9)
*Z*	4	4	2
Radiation type	Mo *K*α	Mo *K*α	Mo *K*α
μ (mm^−1^)	0.11	0.11	0.11
Crystal size (mm)	0.78 × 0.37 × 0.14	0.65 × 0.55 × 0.40	1.30 × 0.58 × 0.22

Data collection			
Diffractometer	Stoe IPDS 2	Stoe IPDS 2	Stoe IPDS 2
Absorption correction	Integration (*X-RED*; Stoe & Cie, 2011 ▸)	Integration (*X-RED*; Stoe & Cie, 2011 ▸)	Integration (*X-RED*; Stoe & Cie, 2011 ▸)
*T*_min_, *T*_max_	0.800, 0.890	0.780, 0.997	0.423, 0.607
No. of measured, independent and observed [*I* > 2σ(*I*)] reflections	10373, 5034, 4398	8812, 4765, 4363	5216, 2527, 2394
*R*_int_	0.026	0.038	0.037
(sin θ/λ)_max_ (Å^−1^)	0.606	0.617	0.595

Refinement			
*R*[*F* ^2^ > 2σ(*F* ^2^)], *wR*(*F* ^2^), *S*	0.032, 0.082, 1.03	0.037, 0.103, 1.03	0.042, 0.112, 1.08
No. of reflections	5034	4765	2527
No. of parameters	458	460	242
No. of restraints	1	401	1
H-atom treatment	H atoms treated by a mixture of independent and constrained refinement	H atoms treated by a mixture of independent and constrained refinement	H atoms treated by a mixture of independent and constrained refinement
Δρ_max_, Δρ_min_ (e Å^−3^)	0.16, −0.18	0.28, −0.20	0.24, −0.20

Computer programs: *X-AREA* and *X-RED* (Stoe & Cie, 2011 ▸), *SHELXS97* (Sheldrick, 2008 ▸), *SHELXL2014* (Sheldrick, 2015 ▸), *ORTEP-3 for Windows* (Farrugia, 2012 ▸), *DIAMOND* (Brandenburg, 2016 ▸) and *publCIF* (Westrip, 2010 ▸).

## supplementary crystallographic information

### (I) [(1R,3R,4R,5R,6S)-4,5-Bis(acetyloxy)-7-oxo-2-oxabicyclo[4.2.0]octan-3-yl]methyl acetate Crystal data

**Table:**

C_14_H_18_O_8_	*F*(000) = 664
*M**_r_* = 314.28	*D*_x_ = 1.374 Mg m^−^^3^
Monoclinic, *P*2_1_	Mo *K*α radiation, λ = 0.71073 Å
*a* = 9.5538 (6) Å	Cell parameters from 15828 reflections
*b* = 8.3655 (3) Å	θ = 2.1–29.6°
*c* = 19.1395 (10) Å	µ = 0.11 mm^−^^1^
β = 96.837 (4)°	*T* = 210 K
*V* = 1518.80 (14) Å^3^	Prism, colourless
*Z* = 4	0.78 × 0.37 × 0.14 mm

### (I) [(1R,3R,4R,5R,6S)-4,5-Bis(acetyloxy)-7-oxo-2-oxabicyclo[4.2.0]octan-3-yl]methyl acetate Data collection

**Table:**

Stoe IPDS 2 diffractometer	5034 independent reflections
Radiation source: sealed X-ray tube	4398 reflections with *I* > 2σ(*I*)
Detector resolution: 6.67 pixels mm^-1^	*R*_int_ = 0.026
rotation method scans	θ_max_ = 25.5°, θ_min_ = 2.1°
Absorption correction: integration (X-RED; Stoe & Cie, 2011)	*h* = −11→11
*T*_min_ = 0.800, *T*_max_ = 0.890	*k* = −10→9
10373 measured reflections	*l* = −23→21

### (I) [(1R,3R,4R,5R,6S)-4,5-Bis(acetyloxy)-7-oxo-2-oxabicyclo[4.2.0]octan-3-yl]methyl acetate Refinement

**Table:**

Refinement on *F*^2^	Secondary atom site location: difference Fourier map
Least-squares matrix: full	Hydrogen site location: mixed
*R*[*F*^2^ > 2σ(*F*^2^)] = 0.032	H atoms treated by a mixture of independent and constrained refinement
*wR*(*F*^2^) = 0.082	*w* = 1/[σ^2^(*F*_o_^2^) + (0.0508*P*)^2^ + 0.0259*P*] where *P* = (*F*_o_^2^ + 2*F*_c_^2^)/3
*S* = 1.03	(Δ/σ)_max_ = 0.056
5034 reflections	Δρ_max_ = 0.16 e Å^−^^3^
458 parameters	Δρ_min_ = −0.18 e Å^−^^3^
1 restraint	Extinction correction: SHELXL2014 (Sheldrick, 2015), Fc^*^=kFc[1+0.001xFc^2^λ^3^/sin(2θ)]^-1/4^
Primary atom site location: structure-invariant direct methods	Extinction coefficient: 0.0067 (15)

### (I) [(1R,3R,4R,5R,6S)-4,5-Bis(acetyloxy)-7-oxo-2-oxabicyclo[4.2.0]octan-3-yl]methyl acetate Special details

**Table:**

Geometry. All esds (except the esd in the dihedral angle between two l.s. planes) are estimated using the full covariance matrix. The cell esds are taken into account individually in the estimation of esds in distances, angles and torsion angles; correlations between esds in cell parameters are only used when they are defined by crystal symmetry. An approximate (isotropic) treatment of cell esds is used for estimating esds involving l.s. planes.

### (I) [(1R,3R,4R,5R,6S)-4,5-Bis(acetyloxy)-7-oxo-2-oxabicyclo[4.2.0]octan-3-yl]methyl acetate Fractional atomic coordinates and isotropic or equivalent isotropic displacement parameters (Å^2^)

**Table:**

	*x*	*y*	*z*	*U*_iso_*/*U*_eq_	
C1A	0.2095 (3)	−0.0487 (3)	0.02643 (14)	0.0311 (5)	
H1A	0.249 (3)	−0.114 (4)	−0.0088 (16)	0.037*	
C2A	0.4221 (3)	0.1880 (4)	−0.01979 (14)	0.0333 (6)	
H21A	0.495 (3)	0.272 (4)	−0.0057 (17)	0.040*	
H22A	0.454 (3)	0.084 (4)	−0.0100 (16)	0.040*	
C3A	0.2922 (2)	0.2231 (3)	0.01673 (14)	0.0300 (5)	
H3A	0.248 (3)	0.322 (4)	0.0017 (16)	0.036*	
C4A	0.3247 (2)	0.2420 (3)	0.09606 (14)	0.0290 (5)	
H4A	0.396 (3)	0.320 (4)	0.1075 (15)	0.035*	
C5A	0.3705 (2)	0.0867 (3)	0.13265 (13)	0.0297 (5)	
H5A	0.369 (3)	0.094 (4)	0.1822 (16)	0.036*	
C6A	0.2818 (2)	−0.0534 (3)	0.10354 (14)	0.0303 (5)	
H6A	0.329 (3)	−0.156 (4)	0.1096 (16)	0.036*	
C7A	0.1355 (3)	−0.0826 (3)	0.12591 (15)	0.0363 (6)	
C8A	0.0717 (3)	−0.1125 (4)	0.05072 (16)	0.0411 (7)	
H81A	−0.015 (3)	−0.047 (4)	0.0315 (17)	0.049*	
H82A	0.057 (3)	−0.223 (5)	0.0436 (17)	0.049*	
C9A	0.3597 (3)	0.0744 (4)	−0.13316 (13)	0.0341 (6)	
C10A	0.3165 (4)	0.1147 (4)	−0.20872 (16)	0.0542 (8)	
H101	0.3998	0.1261	−0.2327	0.081*	
H102	0.2642	0.2144	−0.2118	0.081*	
H103	0.2575	0.0300	−0.2307	0.081*	
C11A	0.1685 (3)	0.4453 (4)	0.12575 (16)	0.0396 (6)	
C12A	0.0374 (4)	0.4765 (4)	0.15925 (19)	0.0527 (8)	
H121	0.0264	0.3941	0.1939	0.079*	
H122	−0.0436	0.4753	0.1235	0.079*	
H123	0.0443	0.5801	0.1821	0.079*	
C13A	0.6163 (3)	0.0545 (3)	0.17332 (14)	0.0341 (6)	
C14A	0.7591 (3)	0.0614 (4)	0.15023 (16)	0.0432 (7)	
H141	0.7759	0.1678	0.1329	0.065*	
H142	0.7654	−0.0159	0.1130	0.065*	
H143	0.8291	0.0368	0.1897	0.065*	
C1B	0.8060 (3)	0.1601 (3)	0.46463 (14)	0.0342 (6)	
H1B	0.759 (3)	0.085 (4)	0.4973 (16)	0.041*	
C2B	0.5757 (3)	0.3730 (4)	0.50869 (14)	0.0346 (6)	
H21B	0.495 (4)	0.447 (4)	0.4929 (17)	0.042*	
H22B	0.547 (3)	0.270 (4)	0.4971 (17)	0.042*	
C3B	0.7071 (3)	0.4238 (3)	0.47699 (14)	0.0301 (5)	
H3B	0.740 (3)	0.525 (4)	0.4984 (16)	0.036*	
C4B	0.6819 (2)	0.4485 (3)	0.39728 (13)	0.0285 (5)	
H4B	0.606 (3)	0.526 (4)	0.3844 (14)	0.034*	
C5B	0.6479 (2)	0.2944 (3)	0.35714 (14)	0.0302 (5)	
H5B	0.658 (3)	0.314 (4)	0.3089 (17)	0.036*	
C6B	0.7401 (3)	0.1586 (3)	0.38603 (13)	0.0308 (5)	
H6B	0.694 (3)	0.060 (4)	0.3727 (15)	0.037*	
C7B	0.8903 (3)	0.1465 (4)	0.36648 (16)	0.0410 (7)	
C8B	0.9501 (3)	0.1105 (4)	0.44202 (18)	0.0464 (7)	
H81B	1.042 (3)	0.187 (4)	0.4664 (17)	0.056*	
H82B	0.968 (4)	0.000 (5)	0.4508 (19)	0.056*	
C9B	0.6282 (3)	0.2577 (4)	0.62289 (14)	0.0341 (6)	
C10B	0.6537 (4)	0.2963 (4)	0.69939 (16)	0.0535 (8)	
H104	0.7407	0.3563	0.7090	0.080*	
H105	0.6609	0.1979	0.7264	0.080*	
H106	0.5759	0.3597	0.7126	0.080*	
C11B	0.8400 (3)	0.6613 (4)	0.37799 (14)	0.0367 (6)	
C12B	0.9765 (3)	0.6992 (4)	0.35068 (16)	0.0468 (7)	
H124	1.0119	0.6043	0.3296	0.070*	
H125	1.0446	0.7349	0.3892	0.070*	
H126	0.9616	0.7831	0.3156	0.070*	
C13B	0.4024 (3)	0.3092 (3)	0.31491 (15)	0.0361 (6)	
C14B	0.2604 (3)	0.2601 (4)	0.33094 (19)	0.0547 (8)	
H144	0.2188	0.3458	0.3556	0.082*	
H145	0.2686	0.1651	0.3603	0.082*	
H146	0.2009	0.2371	0.2874	0.082*	
O1A	0.18233 (17)	0.1092 (2)	−0.00005 (9)	0.0314 (4)	
O2A	0.3858 (2)	0.2071 (2)	−0.09450 (10)	0.0366 (4)	
O3A	0.3709 (2)	−0.0591 (2)	−0.10975 (11)	0.0420 (5)	
O4A	0.19707 (17)	0.2879 (2)	0.12476 (9)	0.0323 (4)	
O5A	0.2391 (3)	0.5442 (3)	0.10160 (14)	0.0595 (6)	
O6A	0.51487 (16)	0.0650 (2)	0.11827 (9)	0.0331 (4)	
O7A	0.5913 (2)	0.0400 (3)	0.23252 (11)	0.0547 (6)	
O8A	0.0895 (2)	−0.0812 (3)	0.18155 (12)	0.0540 (6)	
O1B	0.82288 (18)	0.3164 (2)	0.49431 (9)	0.0329 (4)	
O2B	0.6021 (2)	0.3904 (2)	0.58421 (10)	0.0400 (4)	
O3B	0.6300 (2)	0.1255 (2)	0.59829 (10)	0.0411 (5)	
O4B	0.81215 (17)	0.5039 (2)	0.37353 (9)	0.0322 (4)	
O5B	0.7642 (2)	0.7555 (3)	0.40164 (14)	0.0577 (6)	
O6B	0.50348 (16)	0.2534 (2)	0.36506 (9)	0.0327 (4)	
O7B	0.4278 (2)	0.3865 (3)	0.26526 (11)	0.0478 (5)	
O8B	0.9393 (2)	0.1651 (4)	0.31262 (12)	0.0636 (7)	

### (I) [(1R,3R,4R,5R,6S)-4,5-Bis(acetyloxy)-7-oxo-2-oxabicyclo[4.2.0]octan-3-yl]methyl acetate Atomic displacement parameters (Å^2^)

**Table:**

	*U*^11^	*U*^22^	*U*^33^	*U*^12^	*U*^13^	*U*^23^
C1A	0.0304 (12)	0.0259 (13)	0.0362 (14)	−0.0017 (10)	0.0006 (10)	−0.0029 (12)
C2A	0.0349 (13)	0.0311 (14)	0.0333 (14)	−0.0036 (11)	0.0017 (10)	−0.0015 (12)
C3A	0.0283 (12)	0.0245 (14)	0.0365 (14)	0.0003 (10)	0.0001 (10)	−0.0003 (11)
C4A	0.0248 (12)	0.0256 (12)	0.0363 (13)	−0.0007 (10)	0.0020 (10)	−0.0049 (11)
C5A	0.0249 (12)	0.0334 (14)	0.0300 (12)	0.0032 (10)	−0.0006 (9)	−0.0037 (11)
C6A	0.0295 (13)	0.0256 (13)	0.0355 (14)	0.0042 (10)	0.0021 (10)	0.0015 (11)
C7A	0.0304 (13)	0.0363 (16)	0.0413 (15)	0.0021 (10)	0.0012 (11)	0.0104 (12)
C8A	0.0326 (14)	0.0413 (17)	0.0478 (17)	−0.0102 (12)	−0.0009 (12)	0.0021 (14)
C9A	0.0320 (13)	0.0373 (16)	0.0336 (13)	0.0021 (11)	0.0070 (10)	−0.0029 (13)
C10A	0.072 (2)	0.056 (2)	0.0346 (15)	0.0015 (16)	0.0057 (14)	−0.0039 (15)
C11A	0.0454 (15)	0.0307 (15)	0.0424 (16)	0.0087 (12)	0.0041 (12)	−0.0049 (13)
C12A	0.0566 (19)	0.0470 (19)	0.057 (2)	0.0182 (14)	0.0155 (15)	−0.0022 (16)
C13A	0.0304 (13)	0.0312 (14)	0.0380 (15)	0.0021 (10)	−0.0067 (10)	−0.0038 (12)
C14A	0.0278 (13)	0.0438 (17)	0.0558 (17)	−0.0030 (12)	−0.0039 (11)	−0.0054 (15)
C1B	0.0345 (13)	0.0293 (13)	0.0369 (14)	0.0036 (10)	−0.0037 (11)	0.0005 (12)
C2B	0.0365 (14)	0.0336 (15)	0.0333 (14)	0.0027 (12)	0.0020 (10)	0.0017 (12)
C3B	0.0323 (13)	0.0238 (13)	0.0334 (14)	−0.0020 (10)	0.0005 (10)	−0.0022 (11)
C4B	0.0246 (12)	0.0265 (13)	0.0343 (13)	0.0004 (10)	0.0023 (9)	0.0025 (11)
C5B	0.0265 (12)	0.0331 (14)	0.0303 (13)	−0.0041 (10)	0.0004 (9)	−0.0005 (11)
C6B	0.0305 (12)	0.0253 (13)	0.0356 (14)	−0.0022 (10)	−0.0006 (10)	−0.0051 (11)
C7B	0.0324 (14)	0.0421 (17)	0.0473 (17)	0.0015 (11)	0.0001 (12)	−0.0137 (13)
C8B	0.0350 (15)	0.0453 (19)	0.0566 (18)	0.0133 (12)	−0.0038 (12)	−0.0096 (15)
C9B	0.0311 (13)	0.0389 (16)	0.0333 (13)	−0.0047 (11)	0.0074 (10)	0.0006 (12)
C10B	0.071 (2)	0.055 (2)	0.0342 (15)	−0.0090 (16)	0.0094 (14)	−0.0037 (15)
C11B	0.0443 (15)	0.0299 (14)	0.0352 (14)	−0.0086 (12)	0.0013 (11)	0.0012 (12)
C12B	0.0500 (16)	0.0489 (18)	0.0429 (16)	−0.0209 (14)	0.0109 (12)	−0.0051 (14)
C13B	0.0318 (14)	0.0347 (15)	0.0388 (15)	0.0015 (10)	−0.0077 (11)	−0.0037 (13)
C14B	0.0304 (15)	0.055 (2)	0.076 (2)	−0.0004 (13)	−0.0061 (14)	0.0057 (18)
O1A	0.0268 (9)	0.0286 (10)	0.0368 (9)	−0.0021 (7)	−0.0050 (7)	−0.0004 (8)
O2A	0.0473 (10)	0.0299 (10)	0.0329 (10)	−0.0014 (8)	0.0060 (7)	−0.0004 (8)
O3A	0.0499 (12)	0.0338 (12)	0.0429 (11)	0.0033 (8)	0.0076 (9)	−0.0037 (9)
O4A	0.0298 (9)	0.0282 (10)	0.0391 (10)	0.0032 (7)	0.0053 (7)	−0.0053 (8)
O5A	0.0665 (14)	0.0283 (11)	0.0885 (17)	0.0045 (10)	0.0293 (12)	0.0019 (12)
O6A	0.0227 (8)	0.0427 (11)	0.0327 (9)	0.0037 (7)	−0.0019 (7)	−0.0046 (9)
O7A	0.0413 (11)	0.0849 (18)	0.0355 (11)	0.0043 (11)	−0.0057 (8)	0.0007 (12)
O8A	0.0421 (11)	0.0744 (18)	0.0471 (13)	0.0039 (10)	0.0124 (9)	0.0110 (12)
O1B	0.0306 (9)	0.0303 (10)	0.0356 (9)	0.0026 (7)	−0.0050 (7)	−0.0037 (8)
O2B	0.0528 (11)	0.0331 (10)	0.0355 (10)	0.0009 (8)	0.0114 (8)	−0.0024 (9)
O3B	0.0534 (12)	0.0322 (12)	0.0376 (10)	−0.0055 (8)	0.0047 (8)	0.0000 (9)
O4B	0.0316 (9)	0.0288 (10)	0.0364 (10)	−0.0056 (7)	0.0049 (7)	−0.0001 (8)
O5B	0.0628 (14)	0.0280 (11)	0.0859 (17)	−0.0037 (10)	0.0241 (12)	−0.0034 (12)
O6B	0.0242 (8)	0.0356 (10)	0.0368 (10)	−0.0036 (7)	−0.0026 (7)	0.0033 (8)
O7B	0.0463 (11)	0.0540 (14)	0.0404 (11)	0.0035 (10)	−0.0056 (9)	0.0062 (11)
O8B	0.0405 (12)	0.100 (2)	0.0519 (14)	−0.0025 (12)	0.0115 (10)	−0.0186 (14)

### (I) [(1R,3R,4R,5R,6S)-4,5-Bis(acetyloxy)-7-oxo-2-oxabicyclo[4.2.0]octan-3-yl]methyl acetate Geometric parameters (Å, º)

**Table:**

C1A—O1A	1.427 (3)	C1B—O1B	1.427 (3)
C1A—C8A	1.543 (4)	C1B—C8B	1.548 (4)
C1A—C6A	1.554 (4)	C1B—C6B	1.560 (4)
C1A—H1A	0.98 (3)	C1B—H1B	1.02 (3)
C2A—O2A	1.440 (3)	C2B—O2B	1.445 (3)
C2A—C3A	1.523 (4)	C2B—C3B	1.519 (4)
C2A—H21A	1.00 (3)	C2B—H21B	1.00 (4)
C2A—H22A	0.93 (3)	C2B—H22B	0.92 (4)
C3A—O1A	1.426 (3)	C3B—O1B	1.433 (3)
C3A—C4A	1.521 (4)	C3B—C4B	1.530 (4)
C3A—H3A	0.96 (3)	C3B—H3B	0.97 (3)
C4A—O4A	1.447 (3)	C4B—O4B	1.451 (3)
C4A—C5A	1.516 (4)	C4B—C5B	1.516 (4)
C4A—H4A	0.95 (3)	C4B—H4B	0.98 (3)
C5A—O6A	1.450 (3)	C5B—O6B	1.447 (3)
C5A—C6A	1.513 (4)	C5B—C6B	1.502 (4)
C5A—H5A	0.95 (3)	C5B—H5B	0.95 (3)
C6A—C7A	1.530 (4)	C6B—C7B	1.529 (4)
C6A—H6A	0.97 (3)	C6B—H6B	0.96 (3)
C7A—O8A	1.199 (4)	C7B—O8B	1.192 (4)
C7A—C8A	1.515 (4)	C7B—C8B	1.520 (5)
C8A—H81A	1.02 (3)	C8B—H81B	1.14 (3)
C8A—H82A	0.94 (4)	C8B—H82B	0.96 (4)
C9A—O3A	1.203 (4)	C9B—O3B	1.203 (3)
C9A—O2A	1.341 (3)	C9B—O2B	1.341 (4)
C9A—C10A	1.494 (4)	C9B—C10B	1.491 (4)
C10A—H101	0.9700	C10B—H104	0.9700
C10A—H102	0.9700	C10B—H105	0.9700
C10A—H103	0.9700	C10B—H106	0.9700
C11A—O5A	1.195 (4)	C11B—O5B	1.195 (4)
C11A—O4A	1.346 (3)	C11B—O4B	1.344 (3)
C11A—C12A	1.497 (4)	C11B—C12B	1.496 (4)
C12A—H121	0.9700	C12B—H124	0.9700
C12A—H122	0.9700	C12B—H125	0.9700
C12A—H123	0.9700	C12B—H126	0.9700
C13A—O7A	1.192 (3)	C13B—O7B	1.198 (3)
C13A—O6A	1.347 (3)	C13B—O6B	1.361 (3)
C13A—C14A	1.484 (4)	C13B—C14B	1.484 (4)
C14A—H141	0.9700	C14B—H144	0.9700
C14A—H142	0.9700	C14B—H145	0.9700
C14A—H143	0.9700	C14B—H146	0.9700
			
O1A—C1A—C8A	107.5 (2)	C8B—C1B—H1B	118.3 (18)
O1A—C1A—C6A	113.7 (2)	C6B—C1B—H1B	115.4 (17)
C8A—C1A—C6A	90.2 (2)	O2B—C2B—C3B	108.5 (2)
O1A—C1A—H1A	110.0 (18)	O2B—C2B—H21B	106.0 (19)
C8A—C1A—H1A	115.4 (18)	C3B—C2B—H21B	110.2 (19)
C6A—C1A—H1A	118.4 (17)	O2B—C2B—H22B	110 (2)
O2A—C2A—C3A	108.8 (2)	C3B—C2B—H22B	113.5 (19)
O2A—C2A—H21A	105.4 (18)	H21B—C2B—H22B	108 (3)
C3A—C2A—H21A	108.4 (18)	O1B—C3B—C2B	112.6 (2)
O2A—C2A—H22A	109.6 (19)	O1B—C3B—C4B	109.9 (2)
C3A—C2A—H22A	110.5 (18)	C2B—C3B—C4B	113.4 (2)
H21A—C2A—H22A	114 (2)	O1B—C3B—H3B	104.3 (18)
O1A—C3A—C4A	110.6 (2)	C2B—C3B—H3B	108.2 (17)
O1A—C3A—C2A	112.7 (2)	C4B—C3B—H3B	108.0 (18)
C4A—C3A—C2A	113.4 (2)	O4B—C4B—C5B	104.65 (19)
O1A—C3A—H3A	103.1 (18)	O4B—C4B—C3B	108.45 (19)
C4A—C3A—H3A	103.9 (18)	C5B—C4B—C3B	112.9 (2)
C2A—C3A—H3A	112.4 (18)	O4B—C4B—H4B	110.4 (17)
O4A—C4A—C5A	105.2 (2)	C5B—C4B—H4B	109.0 (17)
O4A—C4A—C3A	108.99 (18)	C3B—C4B—H4B	111.3 (16)
C5A—C4A—C3A	112.7 (2)	O6B—C5B—C6B	107.9 (2)
O4A—C4A—H4A	110.4 (18)	O6B—C5B—C4B	107.3 (2)
C5A—C4A—H4A	108.6 (18)	C6B—C5B—C4B	112.1 (2)
C3A—C4A—H4A	110.9 (18)	O6B—C5B—H5B	110.4 (17)
O6A—C5A—C4A	104.4 (2)	C6B—C5B—H5B	111.2 (18)
O6A—C5A—C6A	109.7 (2)	C4B—C5B—H5B	107.9 (19)
C4A—C5A—C6A	112.08 (19)	C5B—C6B—C7B	119.1 (2)
O6A—C5A—H5A	108.8 (16)	C5B—C6B—C1B	120.0 (2)
C4A—C5A—H5A	111.6 (18)	C7B—C6B—C1B	87.35 (19)
C6A—C5A—H5A	110.2 (18)	C5B—C6B—H6B	108.8 (18)
C5A—C6A—C7A	120.7 (2)	C7B—C6B—H6B	107.2 (17)
C5A—C6A—C1A	119.7 (2)	C1B—C6B—H6B	112.6 (18)
C7A—C6A—C1A	87.25 (19)	O8B—C7B—C8B	134.9 (3)
C5A—C6A—H6A	114.2 (18)	O8B—C7B—C6B	132.8 (3)
C7A—C6A—H6A	104.6 (18)	C8B—C7B—C6B	92.2 (2)
C1A—C6A—H6A	106.5 (18)	C7B—C8B—C1B	88.1 (2)
O8A—C7A—C8A	134.1 (3)	C7B—C8B—H81B	117.4 (17)
O8A—C7A—C6A	133.7 (3)	C1B—C8B—H81B	113.7 (17)
C8A—C7A—C6A	92.2 (2)	C7B—C8B—H82B	114 (2)
C7A—C8A—C1A	88.15 (19)	C1B—C8B—H82B	111 (2)
C7A—C8A—H81A	118.0 (19)	H81B—C8B—H82B	111 (3)
C1A—C8A—H81A	112.8 (19)	O3B—C9B—O2B	123.7 (2)
C7A—C8A—H82A	110 (2)	O3B—C9B—C10B	125.1 (3)
C1A—C8A—H82A	115 (2)	O2B—C9B—C10B	111.2 (3)
H81A—C8A—H82A	112 (3)	C9B—C10B—H104	109.5
O3A—C9A—O2A	124.1 (2)	C9B—C10B—H105	109.5
O3A—C9A—C10A	124.9 (3)	H104—C10B—H105	109.5
O2A—C9A—C10A	111.0 (3)	C9B—C10B—H106	109.5
C9A—C10A—H101	109.5	H104—C10B—H106	109.5
C9A—C10A—H102	109.5	H105—C10B—H106	109.5
H101—C10A—H102	109.5	O5B—C11B—O4B	123.1 (3)
C9A—C10A—H103	109.5	O5B—C11B—C12B	125.8 (3)
H101—C10A—H103	109.5	O4B—C11B—C12B	111.1 (3)
H102—C10A—H103	109.5	C11B—C12B—H124	109.5
O5A—C11A—O4A	123.2 (3)	C11B—C12B—H125	109.5
O5A—C11A—C12A	125.8 (3)	H124—C12B—H125	109.5
O4A—C11A—C12A	111.0 (3)	C11B—C12B—H126	109.5
C11A—C12A—H121	109.5	H124—C12B—H126	109.5
C11A—C12A—H122	109.5	H125—C12B—H126	109.5
H121—C12A—H122	109.5	O7B—C13B—O6B	123.4 (2)
C11A—C12A—H123	109.5	O7B—C13B—C14B	126.2 (3)
H121—C12A—H123	109.5	O6B—C13B—C14B	110.4 (3)
H122—C12A—H123	109.5	C13B—C14B—H144	109.5
O7A—C13A—O6A	122.9 (2)	C13B—C14B—H145	109.5
O7A—C13A—C14A	125.6 (2)	H144—C14B—H145	109.5
O6A—C13A—C14A	111.4 (2)	C13B—C14B—H146	109.5
C13A—C14A—H141	109.5	H144—C14B—H146	109.5
C13A—C14A—H142	109.5	H145—C14B—H146	109.5
H141—C14A—H142	109.5	C1A—O1A—C3A	116.15 (17)
C13A—C14A—H143	109.5	C9A—O2A—C2A	117.6 (2)
H141—C14A—H143	109.5	C11A—O4A—C4A	116.5 (2)
H142—C14A—H143	109.5	C13A—O6A—C5A	118.15 (19)
O1B—C1B—C8B	107.2 (2)	C1B—O1B—C3B	115.80 (18)
O1B—C1B—C6B	113.9 (2)	C9B—O2B—C2B	117.9 (2)
C8B—C1B—C6B	89.9 (2)	C11B—O4B—C4B	117.6 (2)
O1B—C1B—H1B	110.7 (18)	C13B—O6B—C5B	116.8 (2)
			
O2A—C2A—C3A—O1A	65.9 (3)	C8B—C1B—C6B—C5B	−134.1 (3)
O2A—C2A—C3A—C4A	−167.5 (2)	O1B—C1B—C6B—C7B	97.1 (2)
O1A—C3A—C4A—O4A	−57.0 (3)	C8B—C1B—C6B—C7B	−11.7 (2)
C2A—C3A—C4A—O4A	175.3 (2)	C5B—C6B—C7B—O8B	−42.2 (5)
O1A—C3A—C4A—C5A	59.4 (3)	C1B—C6B—C7B—O8B	−165.4 (4)
C2A—C3A—C4A—C5A	−68.3 (3)	C5B—C6B—C7B—C8B	135.2 (2)
O4A—C4A—C5A—O6A	−165.16 (18)	C1B—C6B—C7B—C8B	11.9 (2)
C3A—C4A—C5A—O6A	76.2 (2)	O8B—C7B—C8B—C1B	165.2 (4)
O4A—C4A—C5A—C6A	76.2 (2)	C6B—C7B—C8B—C1B	−12.0 (2)
C3A—C4A—C5A—C6A	−42.4 (3)	O1B—C1B—C8B—C7B	−103.3 (2)
O6A—C5A—C6A—C7A	165.3 (2)	C6B—C1B—C8B—C7B	11.8 (2)
C4A—C5A—C6A—C7A	−79.2 (3)	C8A—C1A—O1A—C3A	140.7 (2)
O6A—C5A—C6A—C1A	−88.7 (3)	C6A—C1A—O1A—C3A	42.5 (3)
C4A—C5A—C6A—C1A	26.7 (3)	C4A—C3A—O1A—C1A	−60.0 (3)
O1A—C1A—C6A—C5A	−25.8 (3)	C2A—C3A—O1A—C1A	68.1 (3)
C8A—C1A—C6A—C5A	−135.1 (2)	O3A—C9A—O2A—C2A	−3.5 (4)
O1A—C1A—C6A—C7A	98.3 (2)	C10A—C9A—O2A—C2A	177.1 (2)
C8A—C1A—C6A—C7A	−11.0 (2)	C3A—C2A—O2A—C9A	−100.7 (2)
C5A—C6A—C7A—O8A	−45.2 (5)	O5A—C11A—O4A—C4A	3.5 (4)
C1A—C6A—C7A—O8A	−168.5 (4)	C12A—C11A—O4A—C4A	−177.7 (2)
C5A—C6A—C7A—C8A	134.5 (3)	C5A—C4A—O4A—C11A	152.2 (2)
C1A—C6A—C7A—C8A	11.2 (2)	C3A—C4A—O4A—C11A	−86.8 (3)
O8A—C7A—C8A—C1A	168.4 (4)	O7A—C13A—O6A—C5A	10.7 (4)
C6A—C7A—C8A—C1A	−11.3 (2)	C14A—C13A—O6A—C5A	−170.0 (2)
O1A—C1A—C8A—C7A	−104.0 (2)	C4A—C5A—O6A—C13A	121.1 (2)
C6A—C1A—C8A—C7A	11.1 (2)	C6A—C5A—O6A—C13A	−118.6 (2)
O2B—C2B—C3B—O1B	68.0 (3)	C8B—C1B—O1B—C3B	140.4 (2)
O2B—C2B—C3B—C4B	−166.4 (2)	C6B—C1B—O1B—C3B	42.6 (3)
O1B—C3B—C4B—O4B	−55.8 (3)	C2B—C3B—O1B—C1B	67.0 (3)
C2B—C3B—C4B—O4B	177.3 (2)	C4B—C3B—O1B—C1B	−60.3 (3)
O1B—C3B—C4B—C5B	59.8 (3)	O3B—C9B—O2B—C2B	−0.5 (4)
C2B—C3B—C4B—C5B	−67.2 (3)	C10B—C9B—O2B—C2B	179.8 (2)
O4B—C4B—C5B—O6B	−166.29 (19)	C3B—C2B—O2B—C9B	−103.5 (3)
C3B—C4B—C5B—O6B	76.0 (2)	O5B—C11B—O4B—C4B	1.5 (4)
O4B—C4B—C5B—C6B	75.5 (2)	C12B—C11B—O4B—C4B	−179.3 (2)
C3B—C4B—C5B—C6B	−42.3 (3)	C5B—C4B—O4B—C11B	155.0 (2)
O6B—C5B—C6B—C7B	162.9 (2)	C3B—C4B—O4B—C11B	−84.2 (3)
C4B—C5B—C6B—C7B	−79.1 (3)	O7B—C13B—O6B—C5B	1.3 (4)
O6B—C5B—C6B—C1B	−91.9 (3)	C14B—C13B—O6B—C5B	−179.0 (2)
C4B—C5B—C6B—C1B	26.0 (3)	C6B—C5B—O6B—C13B	−147.5 (2)
O1B—C1B—C6B—C5B	−25.3 (3)	C4B—C5B—O6B—C13B	91.5 (3)

### (I) [(1R,3R,4R,5R,6S)-4,5-Bis(acetyloxy)-7-oxo-2-oxabicyclo[4.2.0]octan-3-yl]methyl acetate Hydrogen-bond geometry (Å, º)

**Table:**

*D*—H···*A*	*D*—H	H···*A*	*D*···*A*	*D*—H···*A*
C1*A*—H1*A*···O3*A*	0.98 (3)	2.41 (3)	3.184 (3)	135 (2)
C2*A*—H22*A*···O3*A*	0.93 (3)	2.31 (3)	2.697 (3)	104 (2)
C2*A*—H22*A*···O6*A*	0.93 (3)	2.46 (3)	2.876 (3)	107 (2)
C5*A*—H5*A*···O7*A*	0.95 (3)	2.27 (3)	2.701 (3)	106.8 (19)
C1*B*—H1*B*···O3*B*	1.02 (3)	2.44 (3)	3.237 (3)	134 (2)
C2*B*—H22*B*···O3*B*	0.92 (4)	2.34 (3)	2.699 (3)	103 (2)
C2*B*—H22*B*···O6*B*	0.92 (4)	2.52 (3)	2.929 (3)	108 (2)
C5*B*—H5*B*···O7*B*	0.95 (3)	2.34 (3)	2.688 (3)	101 (2)
C4*A*—H4*A*···O3*A*^i^	0.95 (3)	2.44 (3)	3.333 (3)	157 (2)
C2*B*—H21*B*···O3*B*^ii^	1.00 (4)	2.49 (4)	3.400 (3)	150 (2)
C4*B*—H4*B*···O3*B*^ii^	0.98 (3)	2.46 (3)	3.338 (3)	148 (2)
C10*B*—H104···O8*A*^ii^	0.97	2.55	3.306 (4)	135
C10*B*—H106···O7*A*^ii^	0.97	2.52	3.472 (4)	169
C12*B*—H125···O1*B*^iii^	0.97	2.52	3.476 (3)	167

Symmetry codes: (i) −*x*+1, *y*+1/2, −*z*; (ii) −*x*+1, *y*+1/2, −*z*+1; (iii) −*x*+2, *y*+1/2, −*z*+1.

### (II) (1S,4R,5S,6R)-5-Acetyloxy-7-hydroxyimino-2-oxabicyclo[4.2.0]octan-4-yl acetate Crystal data

**Table:**

C_11_H_15_NO_6_	*F*(000) = 544
*M**_r_* = 257.24	*D*_x_ = 1.392 Mg m^−^^3^
Monoclinic, *P*2_1_	Mo *K*α radiation, λ = 0.71073 Å
*a* = 8.9910 (3) Å	Cell parameters from 16172 reflections
*b* = 9.6231 (5) Å	θ = 2.1–29.6°
*c* = 14.4915 (6) Å	µ = 0.11 mm^−^^1^
β = 101.742 (3)°	*T* = 210 K
*V* = 1227.59 (9) Å^3^	Block, colourless
*Z* = 4	0.65 × 0.55 × 0.40 mm

### (II) (1S,4R,5S,6R)-5-Acetyloxy-7-hydroxyimino-2-oxabicyclo[4.2.0]octan-4-yl acetate Data collection

**Table:**

Stoe IPDS 2 diffractometer	4765 independent reflections
Radiation source: sealed X-ray tube	4363 reflections with *I* > 2σ(*I*)
Detector resolution: 6.67 pixels mm^-1^	*R*_int_ = 0.038
rotation method scans	θ_max_ = 26.0°, θ_min_ = 2.3°
Absorption correction: integration (X-RED; Stoe & Cie, 2011)	*h* = −10→11
*T*_min_ = 0.780, *T*_max_ = 0.997	*k* = −11→11
8812 measured reflections	*l* = −17→17

### (II) (1S,4R,5S,6R)-5-Acetyloxy-7-hydroxyimino-2-oxabicyclo[4.2.0]octan-4-yl acetate Refinement

**Table:**

Refinement on *F*^2^	Primary atom site location: structure-invariant direct methods
Least-squares matrix: full	Secondary atom site location: difference Fourier map
*R*[*F*^2^ > 2σ(*F*^2^)] = 0.037	Hydrogen site location: mixed
*wR*(*F*^2^) = 0.103	H atoms treated by a mixture of independent and constrained refinement
*S* = 1.03	*w* = 1/[σ^2^(*F*_o_^2^) + (0.0759*P*)^2^] where *P* = (*F*_o_^2^ + 2*F*_c_^2^)/3
4765 reflections	(Δ/σ)_max_ < 0.001
460 parameters	Δρ_max_ = 0.28 e Å^−^^3^
401 restraints	Δρ_min_ = −0.20 e Å^−^^3^

### (II) (1S,4R,5S,6R)-5-Acetyloxy-7-hydroxyimino-2-oxabicyclo[4.2.0]octan-4-yl acetate Special details

**Table:**

Geometry. All esds (except the esd in the dihedral angle between two l.s. planes) are estimated using the full covariance matrix. The cell esds are taken into account individually in the estimation of esds in distances, angles and torsion angles; correlations between esds in cell parameters are only used when they are defined by crystal symmetry. An approximate (isotropic) treatment of cell esds is used for estimating esds involving l.s. planes.

### (II) (1S,4R,5S,6R)-5-Acetyloxy-7-hydroxyimino-2-oxabicyclo[4.2.0]octan-4-yl acetate Fractional atomic coordinates and isotropic or equivalent isotropic displacement parameters (Å^2^)

**Table:**

	*x*	*y*	*z*	*U*_iso_*/*U*_eq_	Occ. (<1)
C2A	0.7624 (3)	0.8659 (3)	0.34319 (18)	0.0462 (6)	
C9A	0.6416 (4)	0.8926 (4)	0.3973 (2)	0.0555 (8)	
H91A	0.6843	0.9431	0.4545	0.083*	
H92A	0.6004	0.8048	0.4135	0.083*	
H93A	0.5613	0.9473	0.3592	0.083*	
C10A	1.1829 (3)	1.1956 (3)	0.34588 (17)	0.0360 (5)	
C11A	1.1085 (4)	1.3350 (3)	0.3341 (3)	0.0552 (8)	
H111	1.1087	1.3748	0.3956	0.083*	
H112	1.0047	1.3252	0.2996	0.083*	
H113	1.1639	1.3955	0.2994	0.083*	
C1B	0.2924 (3)	0.3975 (3)	0.00453 (17)	0.0366 (5)	
H1B	0.3671	0.3476	−0.0249	0.044*	
C2B	0.7382 (3)	0.5086 (3)	0.16720 (18)	0.0399 (6)	
C3B	0.4218 (3)	0.3777 (3)	0.16338 (18)	0.0411 (6)	
H31B	0.5022	0.3167	0.1501	0.049*	
H32B	0.4066	0.3557	0.2268	0.049*	
C4B	0.4739 (3)	0.5290 (3)	0.16135 (16)	0.0346 (5)	
H4B	0.5018	0.5659	0.2263	0.042*	
C5B	0.3464 (3)	0.6151 (3)	0.10397 (16)	0.0333 (5)	
H5B	0.2559	0.6096	0.1329	0.040*	
C6B	0.3074 (3)	0.5587 (3)	0.00420 (16)	0.0331 (5)	
H6B	0.3788	0.5911	−0.0349	0.040*	
C7B	0.1444 (3)	0.5633 (3)	−0.04794 (16)	0.0381 (5)	
C8B	0.1315 (3)	0.4083 (3)	−0.05803 (19)	0.0442 (6)	
H81B	0.1249	0.3750	−0.1227	0.053*	
H82B	0.0515	0.3674	−0.0297	0.053*	
C9B	0.8617 (3)	0.5288 (4)	0.1139 (2)	0.0504 (7)	
H91B	0.8205	0.5727	0.0539	0.076*	
H92B	0.9049	0.4394	0.1031	0.076*	
H93B	0.9401	0.5876	0.1500	0.076*	
C10B	0.3613 (3)	0.8438 (3)	0.16639 (18)	0.0406 (6)	
C11B	0.4184 (3)	0.9870 (3)	0.15495 (19)	0.0435 (6)	
H114	0.3829	1.0175	0.0904	0.065*	
H115	0.5286	0.9868	0.1696	0.065*	
H116	0.3810	1.0498	0.1974	0.065*	
N1B	0.0567 (3)	0.6686 (3)	−0.06732 (15)	0.0438 (5)	
O2A	0.9009 (2)	0.8930 (2)	0.39572 (12)	0.0433 (4)	
O3A	0.7437 (3)	0.8226 (3)	0.26428 (16)	0.0717 (8)	
O5A	1.2974 (2)	1.1641 (2)	0.32135 (13)	0.0438 (4)	
O1A	1.2131 (13)	0.7019 (9)	0.4315 (5)	0.0375 (14)	0.802 (7)
O4A	1.0968 (5)	1.1064 (4)	0.3838 (3)	0.0393 (9)	0.802 (7)
C3A	1.0769 (10)	0.7171 (8)	0.3612 (7)	0.0399 (15)	0.802 (7)
H31A	0.993 (5)	0.657 (5)	0.381 (3)	0.048*	0.802 (7)
H32A	1.112 (5)	0.682 (5)	0.303 (3)	0.048*	0.802 (7)
C4A	1.0257 (6)	0.8686 (7)	0.3488 (4)	0.0357 (12)	0.802 (7)
H4A	0.993 (4)	0.893 (4)	0.284 (3)	0.043*	0.802 (7)
C5A	1.1497 (6)	0.9650 (6)	0.3974 (4)	0.0321 (11)	0.802 (7)
H5A	1.239 (4)	0.957 (4)	0.369 (2)	0.038*	0.802 (7)
C6A	1.1871 (6)	0.9305 (5)	0.5018 (4)	0.0303 (10)	0.802 (7)
H6A	1.109 (4)	0.978 (4)	0.538 (2)	0.036*	0.802 (7)
C7A	1.3507 (5)	0.9418 (5)	0.5534 (3)	0.0335 (9)	0.802 (7)
C8A	1.3628 (6)	0.7904 (5)	0.5800 (3)	0.0415 (10)	0.802 (7)
H81A	1.365 (5)	0.777 (5)	0.648 (3)	0.050*	0.802 (7)
H82A	1.436 (5)	0.737 (5)	0.562 (3)	0.050*	0.802 (7)
C1A	1.2032 (8)	0.7699 (7)	0.5178 (4)	0.0318 (11)	0.802 (7)
H1A	1.127 (5)	0.725 (5)	0.545 (3)	0.038*	0.802 (7)
N1A	1.4390 (4)	1.0471 (5)	0.5612 (3)	0.0389 (8)	0.802 (7)
O6A	1.5842 (3)	1.0113 (3)	0.61381 (17)	0.0462 (8)	0.802 (7)
H61A	1.647 (5)	1.078 (6)	0.601 (3)	0.055*	0.802 (7)
O1C	1.212 (6)	0.692 (4)	0.445 (2)	0.037 (4)	0.198 (7)
O4C	1.1360 (19)	1.1097 (19)	0.4153 (11)	0.035 (3)	0.198 (7)
C3C	1.098 (5)	0.730 (3)	0.365 (3)	0.037 (4)	0.198 (7)
H31C	1.0084	0.6708	0.3638	0.045*	0.198 (7)
H32C	1.1360	0.7110	0.3079	0.045*	0.198 (7)
C4C	1.047 (2)	0.884 (3)	0.3635 (19)	0.035 (3)	0.198 (7)
H4C	1.0289	0.9168	0.2973	0.042*	0.198 (7)
C5C	1.178 (3)	0.964 (2)	0.4187 (17)	0.032 (3)	0.198 (7)
H5C	1.2681	0.9518	0.3896	0.039*	0.198 (7)
C6C	1.217 (3)	0.919 (3)	0.5190 (17)	0.032 (3)	0.198 (7)
H6C	1.1559	0.9715	0.5569	0.039*	0.198 (7)
C7C	1.3738 (17)	0.8967 (19)	0.5741 (12)	0.031 (3)	0.198 (7)
C8C	1.348 (2)	0.752 (2)	0.6025 (15)	0.041 (3)	0.198 (7)
H81C	1.3378	0.7425	0.6683	0.049*	0.198 (7)
H82C	1.4205	0.6844	0.5867	0.049*	0.198 (7)
C1C	1.194 (4)	0.758 (3)	0.530 (2)	0.035 (3)	0.198 (7)
H1C	1.1035	0.7303	0.5548	0.042*	0.198 (7)
N1C	1.4971 (15)	0.9694 (13)	0.5907 (8)	0.038 (3)	0.198 (7)
O6C	1.4713 (16)	1.1029 (15)	0.5499 (10)	0.045 (3)	0.198 (7)
H61C	1.5341	1.1183	0.5163	0.055*	0.198 (7)
O1B	0.2839 (2)	0.3495 (2)	0.09659 (12)	0.0391 (4)	
O2B	0.60131 (18)	0.54196 (19)	0.11464 (11)	0.0353 (4)	
O3B	0.7546 (3)	0.4639 (3)	0.24570 (15)	0.0639 (7)	
O4B	0.3909 (2)	0.75818 (19)	0.09905 (12)	0.0375 (4)	
O5B	0.3004 (3)	0.8057 (3)	0.22778 (17)	0.0681 (7)	
O6B	−0.0881 (2)	0.6224 (3)	−0.11490 (15)	0.0538 (5)	
H62	−0.146 (5)	0.697 (5)	−0.111 (3)	0.065*	

### (II) (1S,4R,5S,6R)-5-Acetyloxy-7-hydroxyimino-2-oxabicyclo[4.2.0]octan-4-yl acetate Atomic displacement parameters (Å^2^)

**Table:**

	*U*^11^	*U*^22^	*U*^33^	*U*^12^	*U*^13^	*U*^23^
C2A	0.0436 (15)	0.0508 (17)	0.0432 (14)	0.0043 (12)	0.0060 (11)	0.0000 (12)
C9A	0.0423 (16)	0.064 (2)	0.0627 (17)	−0.0009 (14)	0.0156 (13)	−0.0046 (15)
C10A	0.0349 (13)	0.0320 (12)	0.0417 (11)	−0.0021 (10)	0.0092 (10)	0.0021 (10)
C11A	0.0509 (17)	0.0357 (16)	0.082 (2)	0.0051 (13)	0.0213 (15)	0.0132 (14)
C1B	0.0394 (13)	0.0332 (13)	0.0373 (11)	0.0040 (10)	0.0081 (10)	0.0000 (9)
C2B	0.0330 (13)	0.0411 (14)	0.0443 (13)	−0.0003 (10)	0.0044 (10)	0.0038 (11)
C3B	0.0369 (13)	0.0408 (15)	0.0430 (12)	−0.0041 (11)	0.0020 (10)	0.0106 (10)
C4B	0.0301 (12)	0.0402 (14)	0.0341 (10)	−0.0018 (10)	0.0077 (9)	0.0030 (9)
C5B	0.0340 (12)	0.0308 (12)	0.0368 (11)	−0.0015 (9)	0.0113 (9)	0.0011 (9)
C6B	0.0337 (12)	0.0330 (12)	0.0334 (10)	0.0046 (10)	0.0085 (9)	0.0031 (9)
C7B	0.0386 (13)	0.0399 (14)	0.0350 (11)	0.0051 (11)	0.0058 (9)	0.0012 (10)
C8B	0.0443 (15)	0.0438 (15)	0.0403 (13)	0.0003 (12)	−0.0016 (11)	−0.0052 (11)
C9B	0.0390 (14)	0.0549 (18)	0.0588 (15)	0.0016 (13)	0.0132 (12)	0.0079 (13)
C10B	0.0456 (15)	0.0361 (14)	0.0426 (12)	0.0038 (11)	0.0147 (11)	−0.0023 (10)
C11B	0.0479 (15)	0.0358 (15)	0.0497 (13)	0.0010 (12)	0.0166 (11)	−0.0014 (11)
N1B	0.0382 (12)	0.0462 (13)	0.0441 (11)	0.0019 (10)	0.0018 (9)	0.0009 (10)
O2A	0.0387 (10)	0.0518 (12)	0.0411 (9)	−0.0010 (8)	0.0125 (7)	−0.0014 (8)
O3A	0.0579 (14)	0.102 (2)	0.0530 (13)	0.0026 (14)	0.0060 (10)	−0.0175 (13)
O5A	0.0452 (11)	0.0352 (10)	0.0552 (10)	−0.0039 (8)	0.0198 (9)	0.0002 (8)
O1A	0.0395 (17)	0.030 (2)	0.045 (2)	0.0031 (16)	0.014 (2)	−0.0052 (19)
O4A	0.034 (2)	0.0297 (14)	0.057 (2)	0.0048 (13)	0.0166 (15)	0.0089 (17)
C3A	0.043 (3)	0.033 (2)	0.043 (2)	−0.008 (2)	0.007 (2)	−0.0079 (17)
C4A	0.037 (2)	0.040 (2)	0.031 (2)	−0.0025 (17)	0.0078 (16)	−0.0053 (16)
C5A	0.033 (2)	0.0275 (17)	0.037 (2)	0.0026 (16)	0.0103 (16)	0.0016 (16)
C6A	0.027 (2)	0.0267 (18)	0.038 (2)	−0.0022 (15)	0.0068 (14)	−0.0044 (15)
C7A	0.0346 (19)	0.031 (2)	0.0357 (17)	−0.0032 (15)	0.0084 (14)	−0.0014 (14)
C8A	0.038 (2)	0.039 (3)	0.045 (2)	−0.0017 (18)	0.0025 (16)	0.0071 (17)
C1A	0.0311 (18)	0.0293 (19)	0.036 (2)	−0.0019 (14)	0.0102 (15)	0.0011 (15)
N1A	0.0386 (19)	0.039 (2)	0.0381 (15)	−0.0032 (17)	0.0047 (12)	0.0015 (16)
O6A	0.0376 (16)	0.0499 (16)	0.0473 (13)	−0.0141 (12)	−0.0001 (11)	0.0078 (11)
O1C	0.038 (6)	0.033 (6)	0.043 (6)	−0.003 (5)	0.017 (5)	−0.002 (5)
O4C	0.034 (6)	0.027 (4)	0.045 (7)	0.001 (5)	0.013 (5)	0.003 (5)
C3C	0.042 (6)	0.034 (6)	0.040 (6)	−0.007 (6)	0.018 (5)	−0.008 (6)
C4C	0.042 (6)	0.034 (6)	0.033 (6)	0.002 (5)	0.015 (5)	−0.001 (5)
C5C	0.034 (5)	0.022 (5)	0.041 (6)	0.001 (5)	0.010 (5)	−0.005 (5)
C6C	0.034 (5)	0.032 (5)	0.033 (5)	0.007 (4)	0.010 (4)	0.001 (4)
C7C	0.027 (4)	0.029 (5)	0.035 (5)	0.000 (4)	0.006 (4)	0.002 (4)
C8C	0.036 (5)	0.037 (6)	0.050 (6)	−0.002 (5)	0.010 (5)	0.007 (5)
C1C	0.038 (5)	0.030 (5)	0.038 (5)	0.003 (5)	0.011 (5)	−0.005 (5)
N1C	0.034 (6)	0.036 (5)	0.041 (5)	0.002 (5)	0.002 (4)	0.002 (4)
O6C	0.041 (7)	0.042 (7)	0.050 (6)	−0.006 (5)	0.004 (4)	0.007 (6)
O1B	0.0358 (9)	0.0391 (10)	0.0412 (9)	−0.0047 (8)	0.0054 (7)	0.0061 (7)
O2B	0.0312 (9)	0.0391 (10)	0.0363 (8)	−0.0004 (7)	0.0082 (7)	0.0039 (7)
O3B	0.0443 (12)	0.096 (2)	0.0504 (11)	0.0114 (12)	0.0078 (9)	0.0217 (12)
O4B	0.0415 (10)	0.0326 (9)	0.0422 (8)	−0.0006 (7)	0.0171 (7)	−0.0004 (7)
O5B	0.1031 (19)	0.0468 (12)	0.0710 (14)	−0.0122 (13)	0.0571 (14)	−0.0118 (11)
O6B	0.0421 (11)	0.0508 (12)	0.0611 (12)	0.0074 (10)	−0.0070 (9)	−0.0028 (10)

### (II) (1S,4R,5S,6R)-5-Acetyloxy-7-hydroxyimino-2-oxabicyclo[4.2.0]octan-4-yl acetate Geometric parameters (Å, º)

**Table:**

C2A—O3A	1.196 (4)	O2A—C4A	1.444 (6)
C2A—O2A	1.346 (3)	O2A—C4C	1.48 (2)
C2A—C9A	1.485 (4)	O1A—C1A	1.430 (6)
C9A—H91A	0.9700	O1A—C3A	1.433 (7)
C9A—H92A	0.9700	O4A—C5A	1.441 (5)
C9A—H93A	0.9700	C3A—C4A	1.528 (7)
C10A—O5A	1.194 (3)	C3A—H31A	1.03 (5)
C10A—O4A	1.345 (5)	C3A—H32A	1.01 (4)
C10A—O4C	1.431 (18)	C4A—C5A	1.510 (6)
C10A—C11A	1.493 (4)	C4A—H4A	0.96 (4)
C11A—H111	0.9700	C5A—C6A	1.518 (6)
C11A—H112	0.9700	C5A—H5A	0.98 (4)
C11A—H113	0.9700	C6A—C7A	1.512 (5)
C1B—O1B	1.429 (3)	C6A—C1A	1.565 (6)
C1B—C8B	1.546 (4)	C6A—H6A	1.06 (4)
C1B—C6B	1.557 (3)	C7A—N1A	1.278 (6)
C1B—H1B	0.9900	C7A—C8A	1.505 (6)
C2B—O3B	1.197 (3)	C8A—C1A	1.544 (6)
C2B—O2B	1.348 (3)	C8A—H81A	0.99 (4)
C2B—C9B	1.489 (4)	C8A—H82A	0.92 (5)
C3B—O1B	1.435 (3)	C1A—H1A	0.96 (4)
C3B—C4B	1.531 (4)	N1A—O6A	1.414 (5)
C3B—H31B	0.9800	O6A—H61A	0.90 (5)
C3B—H32B	0.9800	O1C—C1C	1.43 (2)
C4B—O2B	1.450 (3)	O1C—C3C	1.43 (2)
C4B—C5B	1.518 (3)	O4C—C5C	1.45 (2)
C4B—H4B	0.9900	C3C—C4C	1.55 (2)
C5B—O4B	1.439 (3)	C3C—H31C	0.9800
C5B—C6B	1.517 (3)	C3C—H32C	0.9800
C5B—H5B	0.9900	C4C—C5C	1.50 (2)
C6B—C7B	1.507 (3)	C4C—H4C	0.9900
C6B—H6B	0.9900	C5C—C6C	1.49 (2)
C7B—N1B	1.279 (4)	C5C—H5C	0.9900
C7B—C8B	1.501 (4)	C6C—C7C	1.489 (19)
C8B—H81B	0.9800	C6C—C1C	1.57 (2)
C8B—H82B	0.9800	C6C—H6C	0.9900
C9B—H91B	0.9700	C7C—N1C	1.291 (17)
C9B—H92B	0.9700	C7C—C8C	1.483 (19)
C9B—H93B	0.9700	C8C—C1C	1.56 (2)
C10B—O5B	1.193 (3)	C8C—H81C	0.9800
C10B—O4B	1.345 (3)	C8C—H82C	0.9800
C10B—C11B	1.492 (4)	C1C—H1C	0.9900
C11B—H114	0.9700	N1C—O6C	1.414 (15)
C11B—H115	0.9700	O6C—H61C	0.8300
C11B—H116	0.9700	O6B—H62	0.89 (5)
N1B—O6B	1.415 (3)		
			
O3A—C2A—O2A	122.8 (3)	O1A—C3A—H32A	101 (2)
O3A—C2A—C9A	126.1 (3)	C4A—C3A—H32A	111 (3)
O2A—C2A—C9A	111.1 (2)	H31A—C3A—H32A	114 (3)
C2A—C9A—H91A	109.5	O2A—C4A—C5A	104.6 (4)
C2A—C9A—H92A	109.5	O2A—C4A—C3A	110.2 (6)
H91A—C9A—H92A	109.5	C5A—C4A—C3A	110.8 (4)
C2A—C9A—H93A	109.5	O2A—C4A—H4A	108 (2)
H91A—C9A—H93A	109.5	C5A—C4A—H4A	111 (2)
H92A—C9A—H93A	109.5	C3A—C4A—H4A	112 (2)
O5A—C10A—O4A	124.2 (3)	O4A—C5A—C4A	108.9 (4)
O5A—C10A—O4C	117.3 (7)	O4A—C5A—C6A	110.1 (4)
O5A—C10A—C11A	126.0 (2)	C4A—C5A—C6A	108.8 (4)
O4A—C10A—C11A	109.8 (3)	O4A—C5A—H5A	107 (2)
O4C—C10A—C11A	114.2 (8)	C4A—C5A—H5A	111 (2)
C10A—C11A—H111	109.5	C6A—C5A—H5A	111 (2)
C10A—C11A—H112	109.5	C7A—C6A—C5A	118.2 (4)
H111—C11A—H112	109.5	C7A—C6A—C1A	86.9 (4)
C10A—C11A—H113	109.5	C5A—C6A—C1A	111.0 (4)
H111—C11A—H113	109.5	C7A—C6A—H6A	113.5 (19)
H112—C11A—H113	109.5	C5A—C6A—H6A	111.4 (19)
O1B—C1B—C8B	110.4 (2)	C1A—C6A—H6A	114 (2)
O1B—C1B—C6B	110.25 (19)	N1A—C7A—C8A	137.2 (4)
C8B—C1B—C6B	90.1 (2)	N1A—C7A—C6A	128.7 (4)
O1B—C1B—H1B	114.5	C8A—C7A—C6A	94.0 (3)
C8B—C1B—H1B	114.5	C7A—C8A—C1A	87.9 (3)
C6B—C1B—H1B	114.5	C7A—C8A—H81A	112 (3)
O3B—C2B—O2B	123.0 (2)	C1A—C8A—H81A	113 (3)
O3B—C2B—C9B	125.4 (2)	C7A—C8A—H82A	119 (3)
O2B—C2B—C9B	111.5 (2)	C1A—C8A—H82A	113 (3)
O1B—C3B—C4B	113.1 (2)	H81A—C8A—H82A	110 (4)
O1B—C3B—H31B	109.0	O1A—C1A—C8A	110.7 (6)
C4B—C3B—H31B	109.0	O1A—C1A—C6A	110.1 (5)
O1B—C3B—H32B	109.0	C8A—C1A—C6A	90.4 (4)
C4B—C3B—H32B	109.0	O1A—C1A—H1A	109 (2)
H31B—C3B—H32B	107.8	C8A—C1A—H1A	118 (2)
O2B—C4B—C5B	106.10 (18)	C6A—C1A—H1A	117 (2)
O2B—C4B—C3B	111.4 (2)	C7A—N1A—O6A	110.1 (4)
C5B—C4B—C3B	109.3 (2)	N1A—O6A—H61A	105 (3)
O2B—C4B—H4B	110.0	C1C—O1C—C3C	114 (3)
C5B—C4B—H4B	110.0	C10A—O4C—C5C	117.9 (16)
C3B—C4B—H4B	110.0	O1C—C3C—C4C	115 (2)
O4B—C5B—C6B	107.93 (19)	O1C—C3C—H31C	108.6
O4B—C5B—C4B	111.3 (2)	C4C—C3C—H31C	108.6
C6B—C5B—C4B	109.2 (2)	O1C—C3C—H32C	108.6
O4B—C5B—H5B	109.5	C4C—C3C—H32C	108.6
C6B—C5B—H5B	109.5	H31C—C3C—H32C	107.6
C4B—C5B—H5B	109.5	O2A—C4C—C5C	117.1 (19)
C7B—C6B—C5B	118.7 (2)	O2A—C4C—C3C	109 (2)
C7B—C6B—C1B	87.4 (2)	C5C—C4C—C3C	106.5 (19)
C5B—C6B—C1B	110.9 (2)	O2A—C4C—H4C	107.9
C7B—C6B—H6B	112.5	C5C—C4C—H4C	107.9
C5B—C6B—H6B	112.5	C3C—C4C—H4C	107.9
C1B—C6B—H6B	112.5	O4C—C5C—C6C	108.9 (17)
N1B—C7B—C8B	137.2 (3)	O4C—C5C—C4C	107.7 (17)
N1B—C7B—C6B	128.8 (3)	C6C—C5C—C4C	111.8 (19)
C8B—C7B—C6B	93.9 (2)	O4C—C5C—H5C	109.5
C7B—C8B—C1B	88.0 (2)	C6C—C5C—H5C	109.5
C7B—C8B—H81B	114.0	C4C—C5C—H5C	109.5
C1B—C8B—H81B	114.0	C7C—C6C—C5C	125 (2)
C7B—C8B—H82B	114.0	C7C—C6C—C1C	86.0 (14)
C1B—C8B—H82B	114.0	C5C—C6C—C1C	112.5 (17)
H81B—C8B—H82B	111.2	C7C—C6C—H6C	110.3
C2B—C9B—H91B	109.5	C5C—C6C—H6C	110.3
C2B—C9B—H92B	109.5	C1C—C6C—H6C	110.3
H91B—C9B—H92B	109.5	N1C—C7C—C8C	129.3 (16)
C2B—C9B—H93B	109.5	N1C—C7C—C6C	134.9 (18)
H91B—C9B—H93B	109.5	C8C—C7C—C6C	95.7 (13)
H92B—C9B—H93B	109.5	C7C—C8C—C1C	86.9 (14)
O5B—C10B—O4B	122.8 (3)	C7C—C8C—H81C	114.2
O5B—C10B—C11B	126.0 (2)	C1C—C8C—H81C	114.2
O4B—C10B—C11B	111.2 (2)	C7C—C8C—H82C	114.2
C10B—C11B—H114	109.5	C1C—C8C—H82C	114.2
C10B—C11B—H115	109.5	H81C—C8C—H82C	111.3
H114—C11B—H115	109.5	O1C—C1C—C8C	109 (2)
C10B—C11B—H116	109.5	O1C—C1C—C6C	108 (2)
H114—C11B—H116	109.5	C8C—C1C—C6C	89.5 (15)
H115—C11B—H116	109.5	O1C—C1C—H1C	115.8
C7B—N1B—O6B	108.9 (2)	C8C—C1C—H1C	115.8
C2A—O2A—C4A	114.9 (3)	C6C—C1C—H1C	115.8
C2A—O2A—C4C	125.9 (10)	C7C—N1C—O6C	110.7 (14)
C1A—O1A—C3A	112.3 (7)	N1C—O6C—H61C	109.5
C10A—O4A—C5A	117.2 (4)	C1B—O1B—C3B	111.43 (19)
O1A—C3A—C4A	111.9 (5)	C2B—O2B—C4B	115.65 (17)
O1A—C3A—H31A	108 (2)	C10B—O4B—C5B	116.91 (19)
C4A—C3A—H31A	110 (2)	N1B—O6B—H62	102 (3)
			
O1B—C3B—C4B—O2B	−104.5 (2)	C7A—C8A—C1A—O1A	105.3 (5)
O1B—C3B—C4B—C5B	12.4 (3)	C7A—C8A—C1A—C6A	−6.4 (4)
O2B—C4B—C5B—O4B	−59.1 (2)	C7A—C6A—C1A—O1A	−105.9 (6)
C3B—C4B—C5B—O4B	−179.27 (19)	C5A—C6A—C1A—O1A	13.3 (7)
O2B—C4B—C5B—C6B	60.0 (3)	C7A—C6A—C1A—C8A	6.4 (4)
C3B—C4B—C5B—C6B	−60.2 (3)	C5A—C6A—C1A—C8A	125.5 (4)
O4B—C5B—C6B—C7B	−93.5 (3)	C8A—C7A—N1A—O6A	−4.5 (7)
C4B—C5B—C6B—C7B	145.4 (2)	C6A—C7A—N1A—O6A	−178.6 (4)
O4B—C5B—C6B—C1B	167.6 (2)	O5A—C10A—O4C—C5C	−29.0 (15)
C4B—C5B—C6B—C1B	46.5 (3)	C11A—C10A—O4C—C5C	168.1 (11)
O1B—C1B—C6B—C7B	−106.2 (2)	C1C—O1C—C3C—C4C	37 (6)
C8B—C1B—C6B—C7B	5.59 (19)	C2A—O2A—C4C—C5C	159.0 (14)
O1B—C1B—C6B—C5B	13.6 (3)	C2A—O2A—C4C—C3C	−80 (2)
C8B—C1B—C6B—C5B	125.4 (2)	O1C—C3C—C4C—O2A	−101 (4)
C5B—C6B—C7B—N1B	58.0 (4)	O1C—C3C—C4C—C5C	27 (4)
C1B—C6B—C7B—N1B	170.5 (3)	C10A—O4C—C5C—C6C	142.3 (16)
C5B—C6B—C7B—C8B	−118.3 (2)	C10A—O4C—C5C—C4C	−96 (2)
C1B—C6B—C7B—C8B	−5.77 (19)	O2A—C4C—C5C—O4C	−60 (3)
N1B—C7B—C8B—C1B	−169.9 (3)	C3C—C4C—C5C—O4C	178 (2)
C6B—C7B—C8B—C1B	5.8 (2)	O2A—C4C—C5C—C6C	60 (3)
O1B—C1B—C8B—C7B	106.1 (2)	C3C—C4C—C5C—C6C	−63 (3)
C6B—C1B—C8B—C7B	−5.61 (19)	O4C—C5C—C6C—C7C	−104 (2)
C8B—C7B—N1B—O6B	−3.7 (4)	C4C—C5C—C6C—C7C	137 (2)
C6B—C7B—N1B—O6B	−178.3 (2)	O4C—C5C—C6C—C1C	155 (2)
O3A—C2A—O2A—C4A	1.3 (5)	C4C—C5C—C6C—C1C	36 (3)
C9A—C2A—O2A—C4A	179.4 (4)	C5C—C6C—C7C—N1C	52 (3)
O3A—C2A—O2A—C4C	4.7 (15)	C1C—C6C—C7C—N1C	166 (2)
C9A—C2A—O2A—C4C	−177.1 (14)	C5C—C6C—C7C—C8C	−125 (2)
O5A—C10A—O4A—C5A	1.7 (5)	C1C—C6C—C7C—C8C	−10.6 (18)
C11A—C10A—O4A—C5A	178.1 (3)	N1C—C7C—C8C—C1C	−166 (2)
C1A—O1A—C3A—C4A	50.4 (12)	C6C—C7C—C8C—C1C	10.7 (18)
C2A—O2A—C4A—C5A	153.7 (3)	C3C—O1C—C1C—C8C	−161 (3)
C2A—O2A—C4A—C3A	−87.2 (4)	C3C—O1C—C1C—C6C	−65 (4)
O1A—C3A—C4A—O2A	−102.4 (9)	C7C—C8C—C1C—O1C	99 (3)
O1A—C3A—C4A—C5A	12.9 (11)	C7C—C8C—C1C—C6C	−10.0 (17)
C10A—O4A—C5A—C4A	−125.2 (5)	C7C—C6C—C1C—O1C	−100 (3)
C10A—O4A—C5A—C6A	115.6 (4)	C5C—C6C—C1C—O1C	26 (3)
O2A—C4A—C5A—O4A	−61.5 (5)	C7C—C6C—C1C—C8C	10.0 (17)
C3A—C4A—C5A—O4A	179.8 (6)	C5C—C6C—C1C—C8C	136 (2)
O2A—C4A—C5A—C6A	58.5 (5)	C8C—C7C—N1C—O6C	179.7 (17)
C3A—C4A—C5A—C6A	−60.1 (7)	C6C—C7C—N1C—O6C	4 (3)
O4A—C5A—C6A—C7A	−96.8 (5)	C8B—C1B—O1B—C3B	−162.5 (2)
C4A—C5A—C6A—C7A	143.9 (5)	C6B—C1B—O1B—C3B	−64.5 (3)
O4A—C5A—C6A—C1A	165.1 (5)	C4B—C3B—O1B—C1B	50.6 (3)
C4A—C5A—C6A—C1A	45.8 (6)	O3B—C2B—O2B—C4B	3.9 (4)
C5A—C6A—C7A—N1A	57.1 (7)	C9B—C2B—O2B—C4B	−178.5 (2)
C1A—C6A—C7A—N1A	169.4 (5)	C5B—C4B—O2B—C2B	161.3 (2)
C5A—C6A—C7A—C8A	−118.9 (4)	C3B—C4B—O2B—C2B	−79.9 (3)
C1A—C6A—C7A—C8A	−6.6 (4)	O5B—C10B—O4B—C5B	−0.4 (4)
N1A—C7A—C8A—C1A	−168.7 (6)	C11B—C10B—O4B—C5B	177.8 (2)
C6A—C7A—C8A—C1A	6.7 (4)	C6B—C5B—O4B—C10B	149.4 (2)
C3A—O1A—C1A—C8A	−162.8 (8)	C4B—C5B—O4B—C10B	−90.8 (3)
C3A—O1A—C1A—C6A	−64.4 (10)		

### (II) (1S,4R,5S,6R)-5-Acetyloxy-7-hydroxyimino-2-oxabicyclo[4.2.0]octan-4-yl acetate Hydrogen-bond geometry (Å, º)

**Table:**

*D*—H···*A*	*D*—H	H···*A*	*D*···*A*	*D*—H···*A*
C5*A*—H5*A*···O5*A*	0.98 (4)	2.21 (4)	2.691 (6)	109 (3)
C11*B*—H115···O3*A*	0.97	2.65	3.417 (4)	136
C9*A*—H92*A*···N1*A*^i^	0.97	2.54	3.482 (6)	163
C4*B*—H4*B*···O6*A*^i^	0.99	2.64	3.407 (3)	135
C9*A*—H92*A*···O6*C*^i^	0.97	2.15	3.115 (15)	175
C1*C*—H1*C*···O4*C*^i^	0.99	2.56	3.52 (4)	165
C11*A*—H112···O3*B*^ii^	0.97	2.59	3.413 (4)	142
C11*B*—H116···O5*A*^iii^	0.97	2.36	3.312 (3)	169
C3*A*—H32*A*···O5*B*^iv^	1.01 (4)	2.50 (4)	3.176 (12)	124 (3)
C3*C*—H32*C*···O5*B*^iv^	0.98	2.25	3.05 (5)	138
C8*A*—H82*A*···O5*A*^v^	0.92 (5)	2.73 (5)	3.329 (5)	124 (3)
C8*C*—H82*C*···O5*A*^v^	0.98	2.62	3.27 (2)	123
C8*C*—H82*C*···O6*C*^v^	0.98	2.50	3.32 (3)	141
O6*B*—H62···O1*B*^vi^	0.89 (5)	1.96 (5)	2.852 (3)	175 (4)
O6*A*—H61*A*···O1*A*^vii^	0.90 (5)	1.86 (5)	2.757 (9)	175 (5)
C11*A*—H113···O6*A*^vii^	0.97	2.61	3.199 (4)	120
O6*C*—H61*C*···O1*C*^vii^	0.83	2.35	2.96 (5)	131
C8*B*—H81*B*···O3*A*^viii^	0.98	2.62	3.498 (4)	150
C9*B*—H92*B*···N1*B*^viii^	0.97	2.69	3.635 (4)	164
C3*B*—H32*B*···O5*A*^ix^	0.98	2.61	3.430 (3)	142
C8*B*—H82*B*···N1*B*^x^	0.98	2.67	3.569 (4)	152

Symmetry codes: (i) −*x*+2, *y*−1/2, −*z*+1; (ii) *x*, *y*+1, *z*; (iii) *x*−1, *y*, *z*; (iv) *x*+1, *y*, *z*; (v) −*x*+3, *y*−1/2, −*z*+1; (vi) −*x*, *y*+1/2, −*z*; (vii) −*x*+3, *y*+1/2, −*z*+1; (viii) −*x*+1, *y*−1/2, −*z*; (ix) *x*−1, *y*−1, *z*; (x) −*x*, *y*−1/2, −*z*.

### (III) [(3aR,5R,6R,7R,7aS)-6,7-Bis(acetyloxy)-2-oxooctahydropyrano[3,2-b]pyrrol-5-yl]methyl acetate Crystal data

**Table:**

C_14_H_19_NO_8_	*F*(000) = 348
*M**_r_* = 329.30	*D*_x_ = 1.381 Mg m^−^^3^
Monoclinic, *P*2_1_	Mo *K*α radiation, λ = 0.71073 Å
*a* = 7.0784 (5) Å	Cell parameters from 7167 reflections
*b* = 6.1454 (3) Å	θ = 2.2–29.6°
*c* = 18.5176 (12) Å	µ = 0.11 mm^−^^1^
β = 100.476 (5)°	*T* = 210 K
*V* = 792.08 (9) Å^3^	Needle, colourless
*Z* = 2	1.30 × 0.58 × 0.22 mm

### (III) [(3aR,5R,6R,7R,7aS)-6,7-Bis(acetyloxy)-2-oxooctahydropyrano[3,2-b]pyrrol-5-yl]methyl acetate Data collection

**Table:**

Stoe IPDS 2 diffractometer	2527 independent reflections
Radiation source: sealed X-ray tube	2394 reflections with *I* > 2σ(*I*)
Detector resolution: 6.67 pixels mm^-1^	*R*_int_ = 0.037
rotation method scans	θ_max_ = 25.0°, θ_min_ = 2.2°
Absorption correction: integration (X-RED; Stoe & Cie, 2011)	*h* = −8→8
*T*_min_ = 0.423, *T*_max_ = 0.607	*k* = −7→6
5216 measured reflections	*l* = −21→21

### (III) [(3aR,5R,6R,7R,7aS)-6,7-Bis(acetyloxy)-2-oxooctahydropyrano[3,2-b]pyrrol-5-yl]methyl acetate Refinement

**Table:**

Refinement on *F*^2^	Secondary atom site location: difference Fourier map
Least-squares matrix: full	Hydrogen site location: mixed
*R*[*F*^2^ > 2σ(*F*^2^)] = 0.042	H atoms treated by a mixture of independent and constrained refinement
*wR*(*F*^2^) = 0.112	*w* = 1/[σ^2^(*F*_o_^2^) + (0.0762*P*)^2^ + 0.0852*P*] where *P* = (*F*_o_^2^ + 2*F*_c_^2^)/3
*S* = 1.08	(Δ/σ)_max_ < 0.001
2527 reflections	Δρ_max_ = 0.24 e Å^−^^3^
242 parameters	Δρ_min_ = −0.20 e Å^−^^3^
1 restraint	Extinction correction: SHELXL2014 (Sheldrick, 2015), Fc^*^=kFc[1+0.001xFc^2^λ^3^/sin(2θ)]^-1/4^
Primary atom site location: structure-invariant direct methods	Extinction coefficient: 0.036 (8)

### (III) [(3aR,5R,6R,7R,7aS)-6,7-Bis(acetyloxy)-2-oxooctahydropyrano[3,2-b]pyrrol-5-yl]methyl acetate Special details

**Table:**

Geometry. All esds (except the esd in the dihedral angle between two l.s. planes) are estimated using the full covariance matrix. The cell esds are taken into account individually in the estimation of esds in distances, angles and torsion angles; correlations between esds in cell parameters are only used when they are defined by crystal symmetry. An approximate (isotropic) treatment of cell esds is used for estimating esds involving l.s. planes.

### (III) [(3aR,5R,6R,7R,7aS)-6,7-Bis(acetyloxy)-2-oxooctahydropyrano[3,2-b]pyrrol-5-yl]methyl acetate Fractional atomic coordinates and isotropic or equivalent isotropic displacement parameters (Å^2^)

**Table:**

	*x*	*y*	*z*	*U*_iso_*/*U*_eq_	
C1	0.2264 (5)	−0.0182 (6)	0.11653 (18)	0.0440 (8)	
H1A	0.289 (5)	−0.143 (8)	0.099 (2)	0.053*	
H1B	0.093 (6)	−0.026 (7)	0.101 (2)	0.053*	
C2	0.5404 (4)	−0.2592 (5)	0.40378 (15)	0.0332 (6)	
C3	0.5365 (5)	−0.3000 (5)	0.32236 (15)	0.0361 (7)	
H31	0.641 (5)	−0.409 (7)	0.3171 (19)	0.043*	
H32	0.417 (5)	−0.357 (7)	0.3040 (19)	0.043*	
C3A	0.5598 (4)	−0.0733 (5)	0.29284 (14)	0.0321 (6)	
H3A	0.695 (5)	−0.039 (6)	0.3008 (18)	0.038*	
C4	0.2361 (4)	0.2171 (7)	0.01733 (16)	0.0441 (8)	
C5	0.2823 (4)	−0.0230 (5)	0.19876 (15)	0.0360 (7)	
H5	0.220 (5)	−0.150 (7)	0.216 (2)	0.043*	
C6	0.2172 (4)	0.1737 (5)	0.23754 (14)	0.0325 (6)	
H6	0.280 (5)	0.303 (7)	0.2235 (19)	0.039*	
C7	0.2592 (4)	0.1260 (5)	0.31927 (15)	0.0314 (6)	
H7	0.180 (5)	0.008 (7)	0.3260 (19)	0.038*	
C7A	0.4729 (4)	0.0772 (5)	0.34508 (15)	0.0305 (6)	
H7A	0.531 (5)	0.221 (7)	0.3522 (18)	0.037*	
C8	0.3107 (5)	0.4270 (8)	−0.0062 (2)	0.0599 (11)	
H8A	0.4376	0.4543	0.0222	0.090*	
H8B	0.2249	0.5441	0.0017	0.090*	
H8C	0.3181	0.4194	−0.0580	0.090*	
C9	−0.0606 (4)	0.4010 (6)	0.20639 (17)	0.0412 (7)	
C10	−0.2673 (4)	0.3985 (7)	0.17223 (17)	0.0440 (8)	
H10A	−0.3349	0.2953	0.1979	0.066*	
H10B	−0.2808	0.3560	0.1211	0.066*	
H10C	−0.3212	0.5426	0.1753	0.066*	
C11	0.0581 (4)	0.3077 (7)	0.39076 (17)	0.0481 (9)	
C12	0.0451 (5)	0.5056 (8)	0.4357 (2)	0.0629 (12)	
H12A	0.0447	0.6340	0.4052	0.094*	
H12B	0.1546	0.5110	0.4757	0.094*	
H12C	−0.0725	0.5010	0.4556	0.094*	
N1	0.5053 (3)	−0.0481 (4)	0.41225 (13)	0.0342 (6)	
H1	0.489 (5)	0.004 (6)	0.454 (2)	0.041*	
O1	0.4857 (3)	−0.0384 (4)	0.21671 (10)	0.0351 (5)	
O2	0.2915 (3)	0.1846 (4)	0.08939 (11)	0.0440 (6)	
O3	0.1378 (4)	0.0922 (6)	−0.02227 (13)	0.0659 (8)	
O4	0.0141 (3)	0.1977 (4)	0.21128 (11)	0.0365 (5)	
O5	0.0300 (4)	0.5594 (5)	0.2283 (2)	0.0696 (9)	
O6	0.2180 (3)	0.3089 (3)	0.36183 (11)	0.0362 (5)	
O7	−0.0571 (4)	0.1649 (7)	0.38027 (18)	0.0869 (12)	
O8	0.5719 (3)	−0.3971 (4)	0.45189 (12)	0.0438 (6)	

### (III) [(3aR,5R,6R,7R,7aS)-6,7-Bis(acetyloxy)-2-oxooctahydropyrano[3,2-b]pyrrol-5-yl]methyl acetate Atomic displacement parameters (Å^2^)

**Table:**

	*U*^11^	*U*^22^	*U*^33^	*U*^12^	*U*^13^	*U*^23^
C1	0.0484 (18)	0.0467 (19)	0.0353 (15)	−0.0139 (16)	0.0038 (13)	−0.0056 (14)
C2	0.0313 (13)	0.0386 (16)	0.0301 (13)	−0.0015 (12)	0.0065 (10)	0.0012 (13)
C3	0.0471 (16)	0.0328 (16)	0.0292 (14)	0.0004 (14)	0.0091 (12)	0.0001 (13)
C3A	0.0347 (14)	0.0350 (17)	0.0271 (13)	−0.0037 (12)	0.0071 (11)	0.0003 (12)
C4	0.0360 (14)	0.069 (2)	0.0283 (14)	0.0077 (17)	0.0076 (12)	0.0025 (17)
C5	0.0386 (15)	0.0381 (16)	0.0308 (14)	−0.0116 (13)	0.0053 (11)	−0.0002 (13)
C6	0.0289 (13)	0.0353 (15)	0.0318 (14)	−0.0087 (12)	0.0017 (10)	0.0025 (13)
C7	0.0312 (13)	0.0323 (15)	0.0302 (13)	−0.0085 (12)	0.0047 (11)	−0.0016 (12)
C7A	0.0329 (13)	0.0291 (15)	0.0293 (14)	−0.0040 (12)	0.0051 (11)	−0.0003 (12)
C8	0.0513 (19)	0.081 (3)	0.0464 (18)	0.004 (2)	0.0067 (15)	0.024 (2)
C9	0.0403 (15)	0.0403 (19)	0.0406 (15)	−0.0085 (15)	0.0009 (12)	0.0105 (14)
C10	0.0374 (15)	0.053 (2)	0.0393 (15)	−0.0030 (15)	0.0019 (12)	0.0064 (16)
C11	0.0281 (14)	0.078 (3)	0.0381 (16)	−0.0025 (16)	0.0043 (11)	−0.0119 (18)
C12	0.0428 (18)	0.088 (3)	0.058 (2)	0.012 (2)	0.0101 (16)	−0.023 (2)
N1	0.0395 (13)	0.0408 (14)	0.0221 (10)	0.0015 (11)	0.0050 (9)	−0.0040 (11)
O1	0.0387 (10)	0.0426 (12)	0.0253 (9)	−0.0048 (9)	0.0091 (8)	0.0029 (9)
O2	0.0474 (12)	0.0536 (14)	0.0289 (10)	−0.0081 (11)	0.0014 (8)	0.0049 (10)
O3	0.0741 (18)	0.091 (2)	0.0304 (11)	−0.0107 (18)	0.0047 (12)	−0.0064 (14)
O4	0.0304 (10)	0.0379 (12)	0.0382 (10)	−0.0091 (9)	−0.0014 (8)	0.0030 (9)
O5	0.0530 (15)	0.0380 (15)	0.108 (2)	−0.0110 (13)	−0.0120 (15)	0.0076 (16)
O6	0.0329 (10)	0.0385 (12)	0.0382 (11)	−0.0004 (9)	0.0092 (8)	−0.0028 (9)
O7	0.0524 (15)	0.128 (3)	0.090 (2)	−0.0434 (19)	0.0389 (15)	−0.054 (2)
O8	0.0492 (12)	0.0491 (14)	0.0346 (11)	0.0065 (11)	0.0117 (9)	0.0118 (10)

### (III) [(3aR,5R,6R,7R,7aS)-6,7-Bis(acetyloxy)-2-oxooctahydropyrano[3,2-b]pyrrol-5-yl]methyl acetate Geometric parameters (Å, º)

**Table:**

C1—O2	1.450 (4)	C7—O6	1.433 (3)
C1—C5	1.502 (4)	C7—C7A	1.531 (4)
C1—H1A	0.98 (5)	C7—H7	0.94 (4)
C1—H1B	0.94 (4)	C7A—N1	1.445 (4)
C2—O8	1.221 (4)	C7A—H7A	0.98 (4)
C2—N1	1.335 (4)	C8—H8A	0.9700
C2—C3	1.524 (4)	C8—H8B	0.9700
C3—C3A	1.516 (4)	C8—H8C	0.9700
C3—H31	1.02 (4)	C9—O5	1.196 (4)
C3—H32	0.92 (4)	C9—O4	1.353 (4)
C3A—O1	1.428 (3)	C9—C10	1.484 (4)
C3A—C7A	1.545 (4)	C10—H10A	0.9700
C3A—H3A	0.97 (4)	C10—H10B	0.9700
C4—O3	1.194 (5)	C10—H10C	0.9700
C4—O2	1.336 (4)	C11—O7	1.190 (5)
C4—C8	1.489 (6)	C11—O6	1.338 (4)
C5—O1	1.421 (4)	C11—C12	1.486 (6)
C5—C6	1.519 (4)	C12—H12A	0.9700
C5—H5	0.98 (4)	C12—H12B	0.9700
C6—O4	1.439 (3)	C12—H12C	0.9700
C6—C7	1.517 (4)	N1—H1	0.87 (4)
C6—H6	0.97 (4)		
			
O2—C1—C5	109.1 (3)	C6—C7—H7	106 (2)
O2—C1—H1A	111 (2)	C7A—C7—H7	113 (2)
C5—C1—H1A	106 (2)	N1—C7A—C7	111.5 (2)
O2—C1—H1B	108 (3)	N1—C7A—C3A	101.6 (2)
C5—C1—H1B	112 (2)	C7—C7A—C3A	113.9 (2)
H1A—C1—H1B	111 (4)	N1—C7A—H7A	112 (2)
O8—C2—N1	127.0 (3)	C7—C7A—H7A	104 (2)
O8—C2—C3	125.2 (3)	C3A—C7A—H7A	115 (2)
N1—C2—C3	107.8 (2)	C4—C8—H8A	109.5
C3A—C3—C2	102.8 (2)	C4—C8—H8B	109.5
C3A—C3—H31	116 (2)	H8A—C8—H8B	109.5
C2—C3—H31	109 (2)	C4—C8—H8C	109.5
C3A—C3—H32	112 (3)	H8A—C8—H8C	109.5
C2—C3—H32	106 (2)	H8B—C8—H8C	109.5
H31—C3—H32	110 (3)	O5—C9—O4	123.4 (3)
O1—C3A—C3	116.7 (2)	O5—C9—C10	125.4 (3)
O1—C3A—C7A	114.3 (2)	O4—C9—C10	111.2 (3)
C3—C3A—C7A	104.0 (2)	C9—C10—H10A	109.5
O1—C3A—H3A	107.1 (19)	C9—C10—H10B	109.5
C3—C3A—H3A	108 (2)	H10A—C10—H10B	109.5
C7A—C3A—H3A	106 (2)	C9—C10—H10C	109.5
O3—C4—O2	123.4 (3)	H10A—C10—H10C	109.5
O3—C4—C8	125.1 (3)	H10B—C10—H10C	109.5
O2—C4—C8	111.5 (3)	O7—C11—O6	122.9 (3)
O1—C5—C1	107.9 (2)	O7—C11—C12	125.8 (3)
O1—C5—C6	108.9 (2)	O6—C11—C12	111.3 (3)
C1—C5—C6	114.6 (3)	C11—C12—H12A	109.5
O1—C5—H5	112 (2)	C11—C12—H12B	109.5
C1—C5—H5	107 (2)	H12A—C12—H12B	109.5
C6—C5—H5	107 (2)	C11—C12—H12C	109.5
O4—C6—C7	111.1 (2)	H12A—C12—H12C	109.5
O4—C6—C5	107.0 (2)	H12B—C12—H12C	109.5
C7—C6—C5	107.3 (2)	C2—N1—C7A	114.8 (2)
O4—C6—H6	108 (2)	C2—N1—H1	121 (3)
C7—C6—H6	114 (2)	C7A—N1—H1	123 (3)
C5—C6—H6	109 (2)	C5—O1—C3A	114.56 (19)
O6—C7—C6	112.0 (2)	C4—O2—C1	114.9 (3)
O6—C7—C7A	105.7 (2)	C9—O4—C6	118.1 (2)
C6—C7—C7A	110.6 (2)	C11—O6—C7	119.2 (2)
O6—C7—H7	110 (2)		
			
O8—C2—C3—C3A	−161.5 (3)	C3—C3A—C7A—C7	−91.8 (3)
N1—C2—C3—C3A	18.0 (3)	O8—C2—N1—C7A	−180.0 (3)
C2—C3—C3A—O1	−155.0 (2)	C3—C2—N1—C7A	0.5 (3)
C2—C3—C3A—C7A	−28.1 (3)	C7—C7A—N1—C2	103.3 (3)
O2—C1—C5—O1	−68.2 (3)	C3A—C7A—N1—C2	−18.4 (3)
O2—C1—C5—C6	53.4 (3)	C1—C5—O1—C3A	−172.2 (3)
O1—C5—C6—O4	173.5 (2)	C6—C5—O1—C3A	62.8 (3)
C1—C5—C6—O4	52.5 (3)	C3—C3A—O1—C5	74.8 (3)
O1—C5—C6—C7	−67.3 (3)	C7A—C3A—O1—C5	−46.9 (3)
C1—C5—C6—C7	171.7 (2)	O3—C4—O2—C1	1.2 (5)
O4—C6—C7—O6	−68.3 (3)	C8—C4—O2—C1	−179.3 (3)
C5—C6—C7—O6	175.1 (2)	C5—C1—O2—C4	−174.5 (3)
O4—C6—C7—C7A	174.1 (2)	O5—C9—O4—C6	−6.4 (5)
C5—C6—C7—C7A	57.5 (3)	C10—C9—O4—C6	174.9 (2)
O6—C7—C7A—N1	81.1 (3)	C7—C6—O4—C9	95.5 (3)
C6—C7—C7A—N1	−157.4 (2)	C5—C6—O4—C9	−147.7 (2)
O6—C7—C7A—C3A	−164.6 (2)	O7—C11—O6—C7	−3.3 (5)
C6—C7—C7A—C3A	−43.2 (3)	C12—C11—O6—C7	177.3 (3)
O1—C3A—C7A—N1	156.5 (2)	C6—C7—O6—C11	103.7 (3)
C3—C3A—C7A—N1	28.2 (3)	C7A—C7—O6—C11	−135.7 (3)
O1—C3A—C7A—C7	36.6 (3)		

### (III) [(3aR,5R,6R,7R,7aS)-6,7-Bis(acetyloxy)-2-oxooctahydropyrano[3,2-b]pyrrol-5-yl]methyl acetate Hydrogen-bond geometry (Å, º)

**Table:**

*D*—H···*A*	*D*—H	H···*A*	*D*···*A*	*D*—H···*A*
C7—H7···O7	0.94 (4)	2.32 (3)	2.695 (4)	103 (3)
C3*A*—H3*A*···O7^i^	0.97 (4)	2.42 (4)	3.243 (4)	143 (3)
C5—H5···O5^ii^	0.98 (4)	2.27 (4)	3.230 (4)	167 (3)
C10—H10*A*···O1^iii^	0.97	2.47	3.386 (4)	158
C12—H12*C*···O8^iv^	0.97	2.58	3.468 (4)	152
N1—H1···O8^v^	0.87 (4)	1.96 (4)	2.826 (3)	174 (4)

Symmetry codes: (i) *x*+1, *y*, *z*; (ii) *x*, *y*−1, *z*; (iii) *x*−1, *y*, *z*; (iv) *x*−1, *y*+1, *z*; (v) −*x*+1, *y*+1/2, −*z*+1.
